# Hexosamine biosynthesis drives hemocyanin O-GlcNAcylation to potentiate antibacterial immunity in shrimp

**DOI:** 10.1016/j.jbc.2026.113213

**Published:** 2026-05-28

**Authors:** Qian Feng, Maoshuai Fu, Wei Wang, Tingchu Wu, Junjie Nie, Zhihong Zheng, Yongzhen Zhao, Jude Juventus Aweya, Yueling Zhang

**Affiliations:** 1 Institute of Marine Sciences and Guangdong Provincial Key Laboratory of Marine Biotechnology, Shantou University, Shantou, China; 3 Guangxi Academy of Fishery Sciences, Guangxi Key Laboratory of Aquatic Genetic Breeding and Healthy Aquaculture, Nanning, China; 2 Department of Medical Laboratory and Department of Reproductive Medicine, Luohu Clinical College of Shantou University Medical College, Shantou University, Shantou, China; 4 College of Ocean Food and Biological Engineering, Jimei University, Xiamen, Fujian, China; 5 Department of Food and Human Nutritional Sciences, University of Manitoba, Winnipeg, Manitoba, Canada; 6 The Canadian Centre for Agri-Food Research in Health and Medicine, St Boniface Hospital Albrechtsen Research Centre, Winnipeg, Manitoba, Canada

**Keywords:** O-GlcNAcylation, hemocyanin, antibacterial immunity, post-translational modification, *Penaeus vannamei*, hexosamine biosynthetic pathway, metabolic reprogramming, host-pathogen interaction

## Abstract

Post-translational modifications (PTMs) are key regulators of immune responses; however, their roles in invertebrate immunity remain poorly defined. Here, we show that *Penaeus vannamei* employs O-GlcNAcylation, a dynamic PTM controlled by the hexosamine biosynthetic pathway (HBP), to enhance antibacterial defense. Bacterial infection induces metabolic reprogramming in hemocytes, upregulating HBP enzymes, and promoting O-GlcNAcylation of hemocyanin (PvHMC) through O-GlcNAc transferase (PvOGT). Site-specific modification of the PvHMC large subunit at Thr584 enhances its conformational stability and interaction with bacterial pathogen-associated molecular patterns, including lipopolysaccharide and peptidoglycan, thereby increasing bacterial binding, agglutination, and killing. Disruption of HBP flux or OGT activity reduces hemocyanin O-GlcNAcylation and impairs bacterial clearance, whereas inhibition of O-GlcNAcase enhances O-GlcNAcylation and antibacterial efficacy. Together, these findings identify HBP-driven O-GlcNAcylation as a metabolic-immune regulatory axis in shrimp and establish hemocyanin O-GlcNAcylation as a key mechanism underlying effective innate antibacterial defense, with potential implications for disease control in aquaculture.

Glucose is a critical energy source for the immune system, and its metabolic intermediates serve as essential intermediates for the post-translational modification (PTM) of immune-related proteins. Beyond its direct utilization by immune cells, particularly during acute inflammatory responses (1), approximately 2 to 5% of cellular glucose is diverted through the hexosamine biosynthetic pathway (HBP), a key branch of glucose metabolism (2). In this pathway, glucose is first metabolized to fructose-6-phosphate, which is subsequently converted by the rate-limiting enzyme fructose-6-phosphate amidotransferase (GFAT) into glucosamine-6-phosphate using glutamine as the amino donor, ultimately leading to the synthesis of uridine diphosphate N-acetylglucosamine (UDP-GlcNAc) (3). UDP-GlcNAc serves as the donor substrate for O-linked N-acetylglucosamine (O-GlcNAc) modification of nuclear and cytoplasmic proteins, a reaction catalyzed by O-GlcNAc transferase (OGT) (4). Removal of the modification is mediated by O-GlcNAcase (OGA) (5), establishing a highly dynamic and reversible regulatory system that plays pivotal roles in immune signaling and homeostasis (6, 7).

In vertebrates, increased HBP flux and O-GlcNAcylation have been linked to both antiviral defense and inflammatory responses. For example, macrophages exposed to vesicular stomatitis virus (VSV), influenza A virus, hepatitis C virus, or bacterial lipopolysaccharides (LPS) display enhanced HBP activity and elevated O-GlcNAcylation, which in turn promotes antiviral signaling and the production of inflammatory cytokines (8, 9). In invertebrates such as *Drosophila melanogaster*, *Penaeus vannamei*, and *Caenorhabditis elegans*, emerging evidence suggests that O-GlcNAcylation also serves as a key regulator of innate immunity (10, 11, 12, 13). However, the molecular mechanisms underlying this modification and its immune functional consequences remain poorly characterized in invertebrate species.

O-GlcNAcylation exerts its effects by modifying serine (Ser) and threonine (Thr) residues on key immune signaling proteins, thereby influencing diverse cellular pathways across taxa (8). In mammals, for instance, O-GlcNAcylation of mitochondrial antiviral-signaling protein (MAVS) at Ser366 enhances retinoic acid-inducible gene-I-like receptor signaling and antiviral immunity (14), while modification of sterile alpha motif and HD domain-containing protein 1 (SAMHD1) at Ser93 stabilizes its antiviral function (15). Similarly, O-GlcNAcylation of receptor-interacting protein kinase 3 (RIPK3) at Thr467 regulates inflammatory necroptosis in response to LPS stimulation (16). In *C. elegans*, O-GlcNAcylation promotes survival following *Staphylococcus aureus* infection by upregulating Cullin-1 (CUL-1) and Cullin-3 (CUL-3), which activate protective immune responses (17). Despite these findings, the contribution of O-GlcNAcylation to immune regulation in invertebrates remains largely unexplored.

Immunometabolic regulation, the integration of metabolic pathways with immune responses, is a fundamental component of host defense (18). Invertebrates, including crustaceans, deploy an array of immune proteins, metabolites, and metabolic circuits to enhance their defense mechanisms (19, 20). Hemocyanin, the multifunctional oxygen-transport protein in mollusks and arthropods, has emerged as a central mediator of immune functions in penaeid shrimp (21, 22, 23, 24, 25). Beyond its molecular diversity, which contributes to immune specificity (22, 26), hemocyanin activity is further shaped by a range of PTMs, including glycosylation, phosphorylation, and acetylation (27, 28, 29, 30). Among these PTMs, N- and O-linked glycosylation have been shown to enhance hemocyanin-mediated immune protection in mollusks and crustaceans (28, 31). However, to our knowledge, no invertebrate study has defined an infection-driven immunometabolic mechanism in which site-specific O-GlcNAcylation of hemocyanin regulates pathogen recognition and antibacterial defense.

In this study, we identify such an immunometabolic mechanism in *P. vannamei*. We show that bacterial infection enhances glucose flux through the HBP, increases hemocyanin O-GlcNAcylation, and augments its antibacterial activity. We further identify Thr584 in the high-molecular-weight hemocyanin subunit as a key O-GlcNAcylation site essential for pathogen recognition and immune activation. Substitution of Thr584 significantly reduces hemocyanin O-GlcNAcylation, diminishes its binding to pathogen-associated molecular patterns (PAMPs), including LPS, peptidoglycan (PGN), and outer membrane proteins (OMPs), and impairs its antibacterial function. Together, these findings reveal a previously unrecognized layer of immunometabolic regulation in crustacean host defense and provide broader insight into the mechanisms governing invertebrate immunity.

## Results

### The hexosamine biosynthetic pathway is induced by bacterial infection

**Figure 1:**
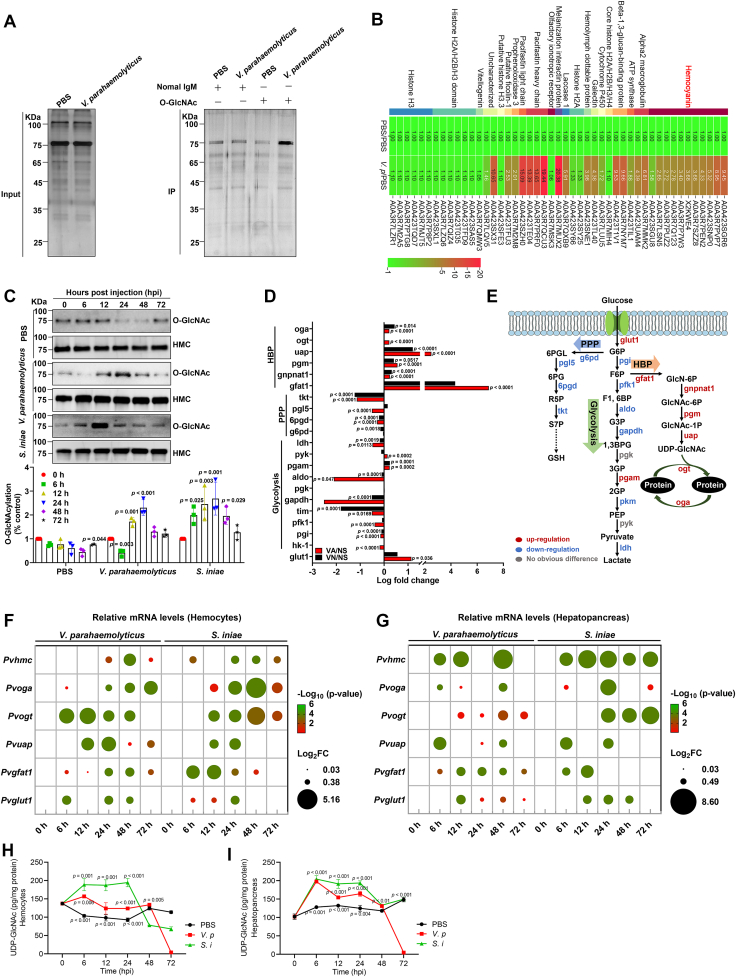
**Bacterial infection enhances hemocyanin O-GlcNAcylation and activates the hexosamine biosynthetic pathway (HBP).***A*, SDS–PAGE analysis of CTD110.6 anti–O-GlcNAc immunoprecipitates from hemolymph of PBS- or *Vibrio parahaemolyticus*-injected *P. vannamei*. *B*, heatmap of candidate O-GlcNAc–associated proteins significantly enriched in CTD110.6 immunoprecipitates following *V. parahaemolyticus* challenge. *C*, immunoblot analysis of size-exclusion chromatography (SEC)-purified PvHMC from hemolymph collected at 0, 6, 12, 24, 48, and 72 h after PBS, *V. parahaemolyticus*, or *S. iniae* injection. O-GlcNAcylated PvHMC was detected using CTD110.6, and total *Pv*HMC using an anti-PvHMC antibody. CTD110.6 signals were normalized to total *Pv*HMC in the same lane and further normalized to the 0 h time point. *D* and *E*, transcriptomic analysis of glucose metabolism pathway genes during *V. parahaemolyticus* (VN) or AHPND *V. parahaemolyticus* (VA) infection. *D*, changes in mRNA abundance (log_10_ scale). *E*, schematic illustration of glycolysis, the pentose phosphate pathway (PPP), and the HBP. *F* and *G*, bubble plots showing relative transcript levels of *Pvhmc*, *Pvglut1*, *Pvuap, Pvogt*, and *Pvoga* in (*F*) hemocytes and (*G*) hepatopancreas at 0, 6, 12, 24, 48, and 72 h following challenge with *V. parahaemolyticus* or *S. iniae*. Bubble size reflects expression changes relative to PBS controls, and bubble color denotes statistical significance as indicated in the legend. *H* and *I*, ELISA-based quantification of UDP-GlcNAc in (*H*) hemocytes and (*I*) hepatopancreas at the indicated time points after *V. parahaemolyticus* or *S. iniae* challenge. Data are presented as mean ± SEM. Statistical significance was assessed by one-way ANOVA with Bonferroni multiple-comparison tests at each time point. Immunoblots and qRT–PCR data are representative of at least three independent experiments.

To investigate the involvement of protein O-GlcNAcylation during bacterial infection, we collected hemolymph from *P. vannamei* injected with either PBS (control) or *Vibrio parahaemolyticus*. After removing N-linked glycans with PNGase F, we performed anti–O-GlcNAc (CTD110.6) immunoprecipitation followed by mass spectrometry (Fig. 1*A*). This approach identified 263 proteins enriched in the CTD110.6 immunoprecipitates (Table S2), which we refer to as candidate O-GlcNAc–associated proteins. Proteins exhibiting increased enrichment in the *V. parahaemolyticus*-challenged group are shown in Figure 1*B*. Notably, hemocyanin (PvHMC), a highly abundant plasma protein with well-characterized immune functions, was one of the most prominently enriched proteins, motivating further characterization.

To determine whether bacterial infection alters hemocyanin O-GlcNAcylation, shrimp were challenged with either *V. parahaemolyticus* (Gram-negative) or *Streptococcus iniae* (Gram-positive). PNGase F–treated hemocyanin purified from plasma was analyzed by Western blot, and the corresponding total protein Coomassie-stained image is shown in Fig. S1*A*. In both infections, hemocyanin O-GlcNAcylation increased significantly, peaking between 12 and 48 h after *V. parahaemolyticus* challenge and at 6 to 48 h after *S. iniae* challenge, compared with PBS-injected controls (Fig. 1*C*). No increase was detected following PBS injection (Fig. 1*C*) or exposure to probiotics (*Bacillus subtilis* or *Enterococcus faecalis*) (Fig. S1*B*), indicating that enhanced O-GlcNAcylation is a specific response to pathogenic infection rather than general immune stimulation.

To evaluate metabolic pathways underlying this response, we reanalyzed our previously published hemocyte transcriptome dataset (GenBank accession: PRJNA385392) from shrimp infected with 2 *V. parahaemolyticus* strains: a virulent strain (VN) and an acute hepatopancreatic necrosis disease (AHPND) strain (VA). Our previous transcriptomic study demonstrated that infection triggers broad transcriptional reprogramming in shrimp, with carbohydrate metabolism identified as one of the major responsive pathways (32). Building on these findings, together with the infection-induced increase in *Pv*HMC O-GlcNAcylation observed in the present study, we performed a focused analysis of genes involved in glucose uptake and the HBP, which governs cellular UDP-GlcNAc production. The analysis revealed significant dysregulation of glucose metabolism genes, including upregulation of *glut1* (glucose transporter 1), *gfat1* (glutamine-fructose-6-phosphate amidotransferase 1), and *uap* (UDP-N-acetylglucosamine pyrophosphorylase) (Fig. 1*D* and Table S3). Since GLUT1 promotes glucose uptake (33) and GFAT1 catalyzes the rate-limiting step of the HBP (34), these transcriptomic changes suggest activation of hexosamine biosynthesis in response to infection.

Consistent with this interpretation, qRT–PCR analysis showed significant induction of *Pvglut1*, *Pvgfat1*, *Pvuap*, and *Pvogt* transcripts following challenges with either *V. parahaemolyticus* or *S. iniae* (Fig. 1, *F* and *G*). *Pvhmc* mRNA levels were also strongly upregulated, particularly from 24 to 72 h post-infection (Fig. 1, *F* and *I*). Supporting these transcriptional changes, ELISA measurements demonstrated that UDP-GlcNAc levels increased from 6 h post-infection and peaked at 24 h in both hemocytes and hepatopancreas (Fig. 1, *H* and *I*). Additionally, total protein O-GlcNAcylation in hemocytes increased markedly after bacterial challenge (Fig. S1, *D* and *E*), whereas PBS injection did not alter O-GlcNAc levels (Fig. S1*C*).

Collectively, these findings indicate that bacterial infection activates the HBP in *P. vannamei*, leading to elevated UDP-O-GlcNAc levels and enhanced O-GlcNAcylation of hemocyanin, suggesting a potentially important mechanism in the shrimp antibacterial response.

### Bacteria induce hemocyanin O-GlcNAcylation *via* the hexosamine biosynthetic pathway

Given that bacterial infection increased PvHMC O-GlcNAcylation (Figs. 1 and S1), we next examined whether this response depends on the glucose uptake–HBP–O-GlcNAc cycling axis. To this end, we used RNAi with two independent siRNAs targeting *Pvglut1*, *Pvgfat1*, *Pvogt*, or *Pvoga*, together with the corresponding pharmacological inhibitors (WZB117, DON, OSMI-4, and Thiamet G).

**Figure 2:**
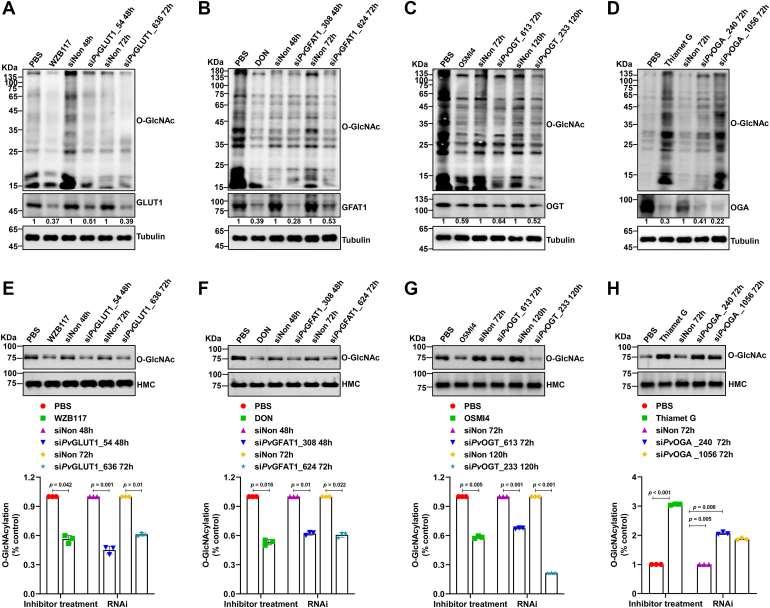
**Inhibition of the hexosamine biosynthetic pathway attenuates hemocyanin O-GlcNAcylation.***A–D*, immunoblot analysis of total O-GlcNAc levels in hemocytes following pharmacological inhibition or siRNA-mediated knockdown of (*A*) *Pv*GLUT1, (*B*) *Pv*GFAT1, (*C*) *Pv*OGT, and (*D*) *Pv*OGA. Blots were probed with anti-O-GlcNAc (CTD110.6), with Tubulin serving as a loading control. *E–G*, immunoblot analysis of *Pv*HMC O-GlcNAcylation in shrimp plasma following pharmacological inhibition or siRNA knockdown of (*E*) *Pv*GLUT1, (*F*) *Pv*GFAT1, or (*G*) *Pv*OGA or *Pv*OGT. *Pv*HMC was purified by SEC and probed with CTD110.6, then reprobed with anti-*Pv*HMC for normalization. Vehicle-treated samples served as controls. Data represent mean + SEM from three independent experiments (n = 3). Statistical significance was determined by one-way ANOVA.

Knockdown of *Pvglut1* (Fig. 2*A*), *Pvgfat1* (Fig. 2*B*), and *Pvogt* (Fig. 2*C*) expression, or pharmacological inhibition of their corresponding protein activities, significantly reduced total O-GlcNAcylation in hemocytes, whereas depletion of *Pvoga* or OGA inhibition caused a clear increase (Fig. 2*D*). Consistently, plasma PvHMC O-GlcNAcylation decreased following suppression of PvGLUT1, PvGFAT1, or PvOGT activity, but increased after *Pvoga* knockdown or Thiamet G treatment (Figs. 2, *E*–*H* and S2*A*). These results confirm that OGT and OGA exert opposing roles in regulating hemocyanin O-GlcNAcylation and that glucose uptake and HBP flux are required for maintaining this modification.

**Figure 3:**
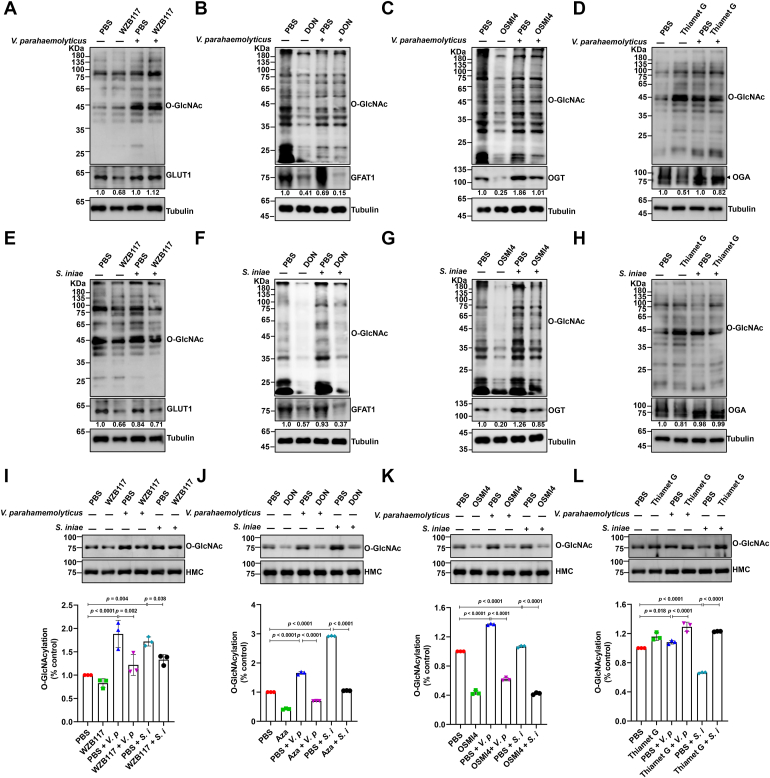
**Pathogenic stimulation activates the HBP to induce hemocyanin O-GlcNAcylation.***A–D*, immunoblot analysis of total O-GlcNAc levels in hemocytes following inhibition of (*A*) *Pv*GLUT1, (*B*) *Pv*GFAT1, (*C*) *Pv*OGT, or (*D*) *Pv*OGA, followed by *V. parahaemolyticus* challenge. *E–H*, immunoblot analysis of total O-GlcNAc levels in hemocytes following inhibition of (*E*) *Pv*GLUT1, (*F*) *Pv*GFAT1, (*G*) *Pv*OGT, or (*H*) *Pv*OGA, followed by *S. iniae* challenge. *I–L*, O-GlcNAcylation levels of *Pv*HMC in shrimp plasma following inhibition of (*I*) *Pv*GLUT1, (*J*) *Pv*GFAT1, (K) *Pv*OGT, or (*L*) *Pv*OGA and subsequent challenge with *V. parahaemolyticus* or *S. iniae*. Data represent mean + SEM from three independent experiments (n = 3). Statistical significance was assessed by one-way ANOVA.

To specifically evaluate how these pathways contribute to infection-induced O-GlcNAcylation, shrimp were pre-treated with WZB117, DON, or OSMI4 before challenge with *V. parahaemolyticus* or *S. iniae*. In all cases, inhibition of glucose uptake, GFAT1 activity, or OGT, significantly decreased total protein O-GlcNAcylation in hemocytes (Fig. 3, *A*–*C*, E, and *F*) and reduced O-GlcNAcylation of plasma hemocyanin (Figs. 3, *I*–*K* and S2*B*) compared with vehicle-treated controls. In contrast, treatment with the OGA-specific inhibitor Thiamet G after bacterial challenge caused a marked increase in O-GlcNAcylation in hemocyte proteins (Fig. 3, *D* and *H*) and in plasma PvHMC (Figs. 3*L* and S2*B*).

Together, these data demonstrate that infection-induced hemocyanin O-GlcNAcylation is driven by activation of the HBP and subsequent modulation of OGT/OGA activity in hemocytes and plasma. This highlights a central role for metabolic reprogramming in shaping the antibacterial immune response of *P. vannamei*.

### *P*vOGT mediates O-GlcNAcylation of hemocyanin in response to bacterial challenge

**Figure 4:**
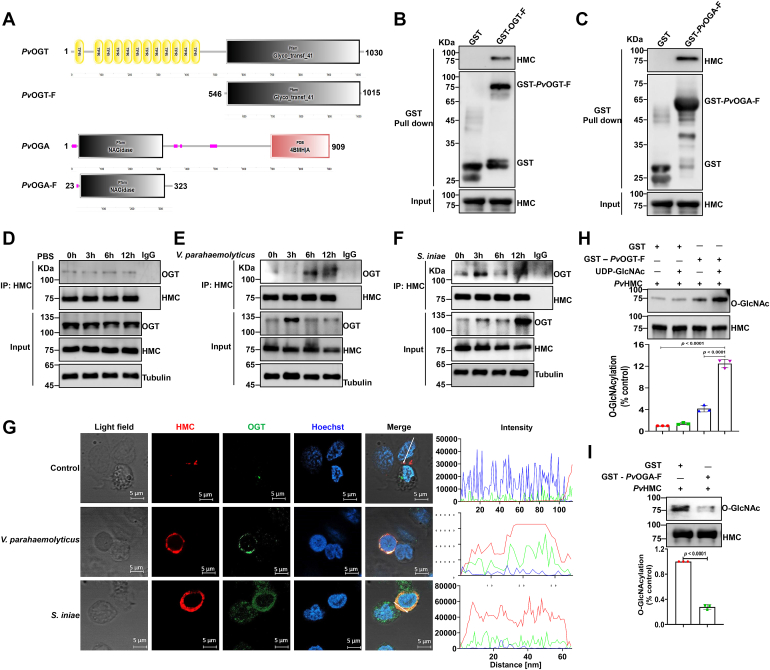
***Pv*OGT mediates hemocyanin O-GlcNAcylation during pathogen infection.***A*, domain organization of *Pv*OGT and *Pv*OGA. *Pv*OGT-F represents a C-terminal catalytic domain mutant, and *Pv*OGA-F represents an N-terminal catalytic domain mutant. *B* and *C*, GST pull-down assays demonstrating interactions between *Pv*HMC and (*B*) GST-*Pv*OGT-F or (*C*) GST-*Pv*OGA-F. The Input/HMC panels in *B* and *C* share the same source image, as both assays used the same PvHMC input sample. *D–F*, *in vivo* immunoprecipitation and immunoblot analysis of hemocytes from sshrimp infected with (*D*) PBS, (*E*) *V. parahaemolyticus*or, (*F*) *S. iniae*, probed with anti-*Pv*HMC. *G*, confocal micro*s*copy of hemocytes mock-infected or infected with *V. parahaemolyticus* or *S. iniae*, probed with anti-*Pv*HMC and anti-OGT. Nuclei were stained with Hoechst 33342. *H*, *In vitro* O-GlcNAcylation assay of PvHMC detected by immunoblotting. *I*, *in vitro* removal of O-GlcNAc from *Pv*HMC assessed by immunoblotting. Data represent mean + SEM from three independent experiments (n = 3). Statistical significance was determined by one-way ANOVA.

Having established that disruption of PvOGT markedly reduces O-GlcNAcylation of both hemocyte proteins and plasma hemocyanin, we next examined whether PvOGT directly interacts with PvHMC and catalyzes its modification during bacterial infection. To assess this, we generated deletion constructs of PvOGT and PvOGA containing only their catalytic domains, which retain O-GlcNAc transferase and O-GlcNAcase activity, respectively (Fig. 4*A*). GST pull-down assays revealed that the catalytic domains of both PvOGT and PvOGA were essential for binding to PvHMC (Fig. 4, *B* and *C*), suggesting that the enzymatic regions of these proteins mediate their association with hemocyanin.

To evaluate this interaction *in vivo*, co-immunoprecipitation analyses were performed in shrimp challenged with *V. parahaemolyticus* or *S. iniae*. In PBS-injected controls, PvOGT - PvHMC interaction was weak or near background (Fig. 4*D*). In contrast, their association increased markedly following bacterial infection with either pathogen (Fig. 4, *E* and *F*), indicating that bacterial challenge promotes PvOGT recruitment to hemocyanin. This infection-dependent association was further supported by immunofluorescence microscopy, which showed clear co-localization of endogenous PvOGT and PvHMC in hemocytes after infection with both *V. parahaemolyticus* and *S. iniae* (Fig. 4*G*).

To directly test whether PvOGT catalyzes hemocyanin O-GlcNAcylation, we performed *in vitro* enzymatic assays. The catalytic domain of PvOGT efficiently transferred O-GlcNAc to PvHMC (Figs. 4*H* and S2, *C* and *D*). Conversely, PvOGA removed O-GlcNAc from PvHMC *in vitro* (Fig. 4*I* and S2, *E* and *F*), confirming that hemocyanin O-GlcNAcylation is dynamically regulated by the opposing activities of OGT and OGA.

Collectively, these results demonstrate that bacterial infection enhances PvOGT association with hemocyanin and promotes PvOGT-mediated O-GlcNAcylation of PvHMC *in vivo*. This dynamic modification likely contributes to the modulation of the antibacterial immune response in *P. vannamei*.

### O-GlcNAcylation enhances the antibacterial activity of *Pv*HMC

**Figure 5:**
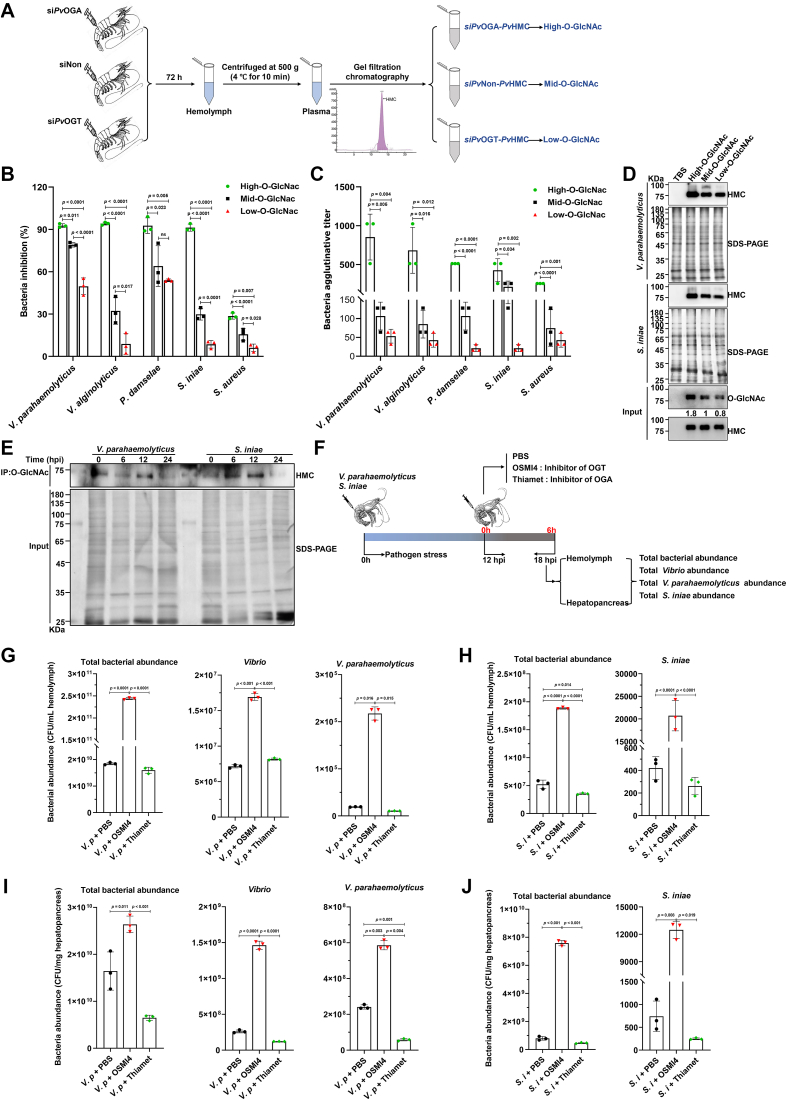
***Pv*OGT-mediated O-GlcNAcylation enhances the antimicrobial activity of *Pv*HMC.***A*, schematic overview of shrimp injections, sample collection, and purification of plasma O-GlcNAcylated *Pv*HMC. *B*, Antibacterial activity of *Pv*HMC samples with high, mid, or low O-GlcNAcylation against *V. parahaemolyticus*, *V. alginolyticus*, *P. damselae*, *S. iniae*, and *S. aureus*. *C*, bacterial agglutination activity of PvHMC with different O-GlcNAcylation levels. *D*, binding of *Pv*HMC to *V. parahaemolyticus* and *S. iniae*, assessed by immunoblotting. *E*, immunoprecipitation and immunoblot analysis of plasma bacteria following infection with *V. parahaemolyticus* or *S. iniae*, probed for O-GlcNAcylation and hemocyanin. *F*, experimental schematic of shrimp injections and pathogen challenge. *G-H*, bacterial abundance in hemolymph following chemical inhibition of *Pv*OGT or *Pv*OGA and subsequent infection with (*G*) *V. parahaemolyticus* or (*H*) *S. iniae*. *I–J*, bacterial abundance in hepatopancreas following chemical inhibition and infection with (*I*) *V. parahaemolyticus* or (*J*) *S. iniae*. Data represent mean ± SEM from three independent experiments. Statistical significance was assessed by one-way ANOVA.

Given the well-established role of hemocyanin (PvHMC) in crustacean antimicrobial immunity (22), we next examined how O-GlcNAcylation influences its antibacterial function in *P. vannamei*. To generate hemocyanin with distinct O-GlcNAcylation states, plasma samples were collected from shrimp treated with siPvOGA (high), siCtrl (medium), or siPvOGT (low) (Figs. 5*A* and S3*F*). These samples were assessed for antibacterial, agglutination, and bacterial-binding activity against three Gram-negative pathogens (*V. parahaemolyticus, Vibrio alginolyticus, Photobacterium damselae*) and two Gram-positive pathogens (*S. iniae, S. aureus*).

Hemocyanin with medium and low O-GlcNAcylation exhibited significantly reduced antibacterial activity (Figs. 5*B* and S3*A*) and diminished agglutination capacity (Figs. 5*C* and S3*B*) compared with the high-O-GlcNAc form. Importantly, O-GlcNAcylation did not affect hemocyanin activity toward probiotic bacteria (*E. faecalis, Pediococcus pentosaceus, B. subtilis*), consistent with previous observations for hemocyanin phosphorylation (Fig. S3*C*).

Interestingly, direct *in vitro* incubation of hemocyanin with bacterial strains induced a significant increase in O-GlcNAcylation when exposed to pathogens (*V. parahaemolyticus, V. alginolyticus, S. iniae*), but not to probiotics (*E. faecalis, P. pentosaceus*) (Fig. S3, *D* and *E*). Correspondingly, hemocyanin with reduced O-GlcNAcylation (mid or low) showed impaired binding affinity toward pathogenic bacteria, whereas high-O-GlcNAc hemocyanin displayed the strongest binding (Figs. 5*D* and S3*F*). This enhanced binding *in vivo* was particularly evident following challenge with *V. parahaemolyticus* and *S. iniae*, with maximal association occurring at 12 hpi for *V. parahaemolyticus* and 6 to 12 hpi for *S. iniae* (Fig. 5*E*).

To further determine the functional importance of hemocyanin O-GlcNAcylation in bacterial clearance, shrimp were challenged with *V. parahaemolyticus* or *S. iniae* and then treated with either the PvOGT inhibitor OSMI-4 or the PvOGA inhibitor Thiamet G (Fig. 5*F*). Inhibition of PvOGT (Fig. S4, *A* and *C*) or PvOGA (Fig. S4, *B* and *D*), respectively, decreased or increased infection-induced O-GlcNAcylation in hemocyte proteins and plasma PvHMC. Functionally, loss of O-GlcNAcylation *via* OGT inhibition resulted in significantly higher bacterial loads, including total bacteria, *Vibrio*, *V. parahaemolyticus*, and *S. iniae*, in both hemolymph (Fig. 5, *G* and *H*) and hepatopancreas (Fig. 5, *I* and *J*). Conversely, increasing O-GlcNAcylation through Thiamet G treatment substantially reduced pathogen load in both tissues (Fig. 5, *G*–*J*).

Consistent results were obtained after inhibition of *Pvglut1* and *Pvgfat1* with WZB117 and DON, respectively, following infection. Both treatments led to pronounced increases in bacterial abundance in hemolymph and hepatopancreas (Fig. S4, *E*–*I*), further supporting the requirement for HBP-driven O-GlcNAcylation during antibacterial defense.

Taken together, these findings demonstrate that O-GlcNAcylation enhances the antibacterial activity of hemocyanin in *P. vannamei*, promoting bacterial agglutination, binding, and clearance. Thus, hemocyanin O-GlcNAcylation represents a key regulatory mechanism underpinning effective innate immune responses during bacterial infection.

### Thr584 is critical for PvOGT-mediated O-GlcNAcylation of PvHMCL

**Figure 6:**
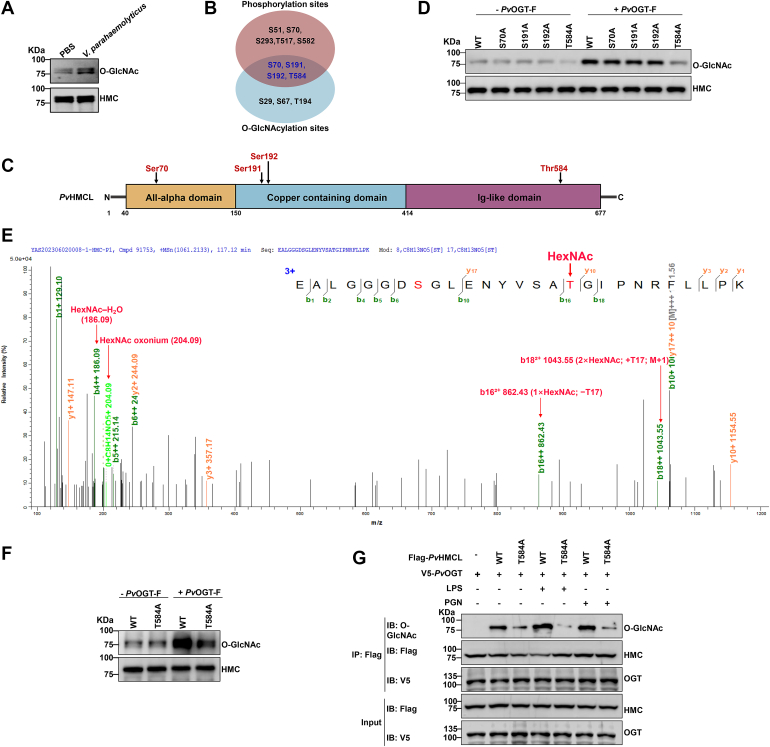
**PvOGT mediates O-GlcNAcylation of *Pv*HMCL at Thr584.***A*, gel-filtration chromatography of hemocyanin isolated from hemolymph of PBS- or *V. parahaemolyticus*-injected shrimp. *B*, identified phosphorylation (*green*) and O-GlcNAcylation (*red*) sites on *Pv*HMCL based on phosphorylation site mass spectrometry (PRIDE: PXD032927) and O-GlcNAcylation site mass spectrometry following *V. parahaemolyticus* stimulation (PRIDE: PXD046737). *C*, overlapping phosphorylation and O-GlcNAcylation sites on *Pv*HMCL. *D*, immunoblot analysis of O-GlcNAcylation of wild-type and mutant *Pv*HMCL-Flag proteins following incubation with recombinant *Pv*OGT-F. *E*, HCD LC–MS/MS spectrum confirming O-GlcNAcylation of PvHMC at Thr584. HexNAc diagnostic oxonium ions (m/z 204.09, 186.09) and site-determining fragment ions localize the modification to T17 (Thr584): b16^2+^ 862.43 (1×HexNAc; −T17) *versus* b18^2+^ 1043.55 (2×HexNAc; +T17; M+1). Other potential site assignments suggested by this PSM could not be confidently localized and are not interpreted. *F*, *in vitro* O-GlcNAcylation assays of wild-type and T584A mutant PvHMCL-Flag. *G*, co-immunoprecipitation and immunoblot analysis of *Drosophila* S2 cells transfected with V5-OGT and Flag-tagged *Pv*HMCL WT or T584A mutant following LPS or PGN stimulation. Data represent three independent experiments.

To identify key O-GlcNAcylation sites on hemocyanin, we analyzed plasma samples from *V. parahaemolyticus*-challenged shrimp and PBS-treated controls for GlcNAcylated PvHMC (Fig. 6*A*). Mass spectrometry revealed seven infection-induced O-GlcNAcylation sites on the large hemocyanin subunit (PvHMCL): Ser29, Ser67, Ser70, Ser191, Ser192, Thr194, and Thr584 (Table S4 and Fig. 6*B*).

*In silico* predictions using O-GlcNAcPRED-DL, NetOGlyc 4.0, and YinOYang 1.2 supported these findings and ranked these residues, particularly Thr584, among the strongest candidates for O-GlcNAc modification (Fig. S5). Comparison with our previous phosphoproteomic dataset (PRIDE: PXD032927) further showed that Ser70, Ser191, Ser192, and Thr584 are dual-modification sites, indicating potential crosstalk between O-GlcNAcylation and phosphorylation (Fig. 6*B*).

To assess the functional significance of these residues, we generated four PvHMCL point mutants (S70A, S191A, S192A, and T584A) (Fig. 6*C*). Mutant and wild-type proteins expressed in HEK 293T cells were purified and subjected to *in vitro* O-GlcNAcylation assays using recombinant PvOGT-F. While the S70A, S191A, and S192A mutants showed O-GlcNAcylation levels comparable to wild-type PvHMCL, the T584A mutant exhibited almost no detectable modification (Fig. 6*D*), identifying Thr584 as a critical O-GlcNAcylation site (Fig. 6*E*).

Together, these results establish Thr584 as an indispensable O-GlcNAcylation site on PvHMCL and a key determinant of PvOGT-dependent regulation of hemocyanin during the immune response in *P. vannamei*.

Direct biochemical validation confirmed that PvOGT-F could no longer modify the T584A mutant *in vitro* (Fig. 6*F*). To test the relevance of this site in a cellular context, *Drosophila* S2 cells were co-transfected with plasmids encoding wild-type PvHMCL (WT-Flag) or the T584A mutant (T584A-Flag) together with PvOGT (OGT-V5). Following stimulation with LPS or PGN, strong O-GlcNAcylation was observed on wild-type PvHMCL but was absent in the T584A mutant (Fig. 6*G*), demonstrating that Thr584 is essential for PvOGT-mediated modification during immune stimulation.

Together, these results establish Thr584 as an indispensable O-GlcNAcylation site on PvHMCL and a key determinant of PvOGT-dependent regulation of hemocyanin during the immune response in *P. vannamei*.

### O-GlcNAcylation of PvHMCL at Thr584 enhances its antibacterial activity

**Figure 7:**
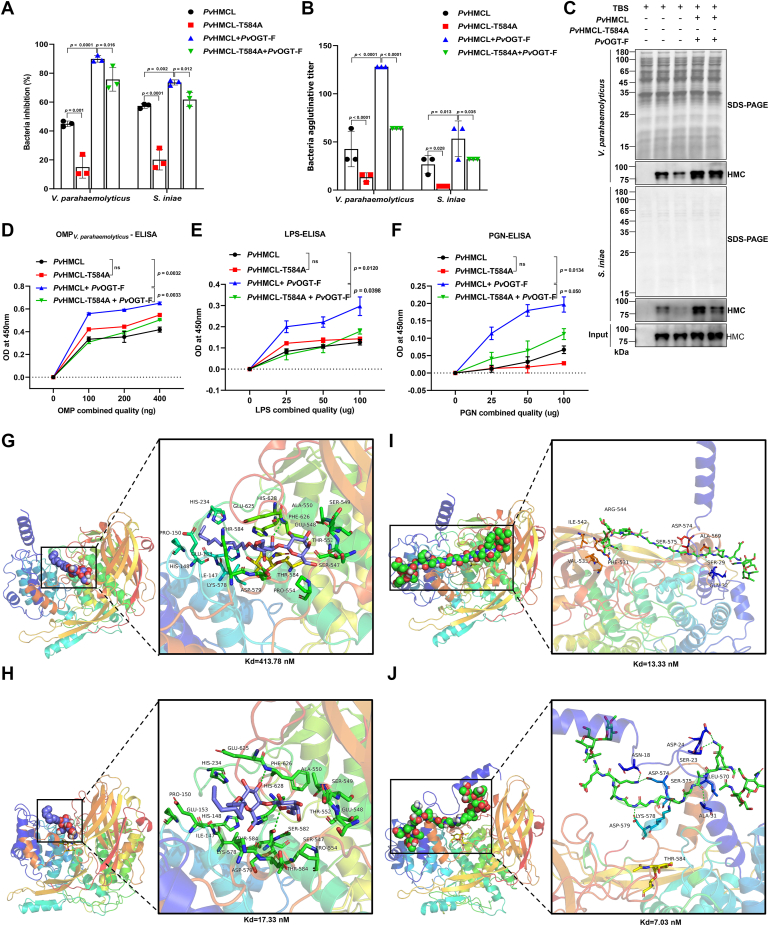
**O-GlcNAcylation of *Pv*HMCL at Thr584 is essential for antibacterial activity.***A*, bacterial inhibition assay using *Pv*HMCL, *Pv*HMCL-T584A, *Pv*HMCL+*Pv*OGT-F, and *Pv*HMCL-T584A+*Pv*OGT-F (100 μg/ml) against *V. parahaemolyticus* and *S. iniae*. *B*, bacterial agglutination assays with *V. parahaemolyticus* and *S. iniae* in the presence or absence of calcium. *C*, bacterial binding activity of *Pv*HMCL variants assessed by immunoblotting. *D*, ELISA analysis of *Pv*HMCL binding to *V. parahaemolyticus* outer membrane proteins (OMPs). *E* and *F*, ELISA analysis of *Pv*HMCL binding to (*E*) LPS and (*F*) PGN. *G–I*, molecular docking of (*G* and *H*) LPS and (*I*–*J*) PGN with *Pv*HMCL or O-GlcNAcylated *Pv*HMCL (GlcNAc-T584). Key residues are shown as *green sticks*, hydrogen bonds as *dashed lines*, LPS in *purple*, and PGN in *green*. Data represent mean + SEM from three independent experiments with biological replicates (n = 3). Statistical significance was assessed by One-way ANOVA.

To determine the functional significance of Thr584 O-GlcNAcylation in the antibacterial activity of PvHMCL, we performed a series of *in vitro* assays using wild-type PvHMCL (with or without PvOGT-F treatment) and the PvHMCL-T584A mutant (with or without PvOGT-F treatment). Antibacterial assays against *V. parahaemolyticus* and *S. iniae* showed that wild-type PvHMCL exhibited significantly stronger bactericidal activity (Figs. 7*A* and S6*A*), bacterial agglutination (Figs. 7*B* and S6*B*), and bacterial binding capacity (Fig. 7*C*) compared with the T584A mutant. Notably, PvOGT-F treatment further augmented all antibacterial functions of wild-type PvHMCL but did not enhance the activity of the T584A mutant, underscoring the specific requirement of Thr584 for O-GlcNAc-mediated functional enhancement.

PvOGT-F-treated wild-type PvHMCL also showed markedly increased binding to bacterial OMPs and to key PAMPs, including LPS and PGN, in comparison with untreated PvHMCL and with the T584A mutant (Figs. 7, *D*–*F* and S6*C*).

To further elucidate the molecular basis of these effects, we performed molecular docking analyses using an AlphaFold2-predicted PvHMCL structure (Fig. S6*D*). Model reliability was supported by Ramachandran analysis, which indicated that 99% of residues resided in favored conformations (Fig. S6*E*) and by subsequent structural refinement using the Amber14SB force field (Fig. S6, *F* and *G*). Docking simulations with LPS lipid A (35) and PGN core structures (36) revealed that wild-type PvHMCL formed a hydrogen bond (2.86 Å) and van der Waals interactions with Phe626 (Fig. 7*G*). Simulated O-GlcNAcylation at Thr584 (PvHMCL-O-GlcNAcT584) introduced an additional hydrogen bond with Ser582 (2.4 Å) while maintaining the interaction with Phe626 (Fig. 7*H*), thereby reinforcing ligand binding. Consistent with these structural observations, the dissociation constant (Kd) for LPS binding decreased dramatically from 413.78 nmol/L in wild-type PvHMCL to 17.33 nmol/L in the O-GlcNAcylated form (Table S5).

For PGN binding, wild-type PvHMCL established hydrogen bonds with Arg544, Ser575, and Asp574 (Figs. 7*I* and S6*H*). O-GlcNAcylation at Thr584 further stabilized these interactions by introducing additional hydrogen bonds (Figs. 7*J* and S6*I*), resulting in an improved binding affinity (Kd reduced from 13.33 nmol/L in the wild-type to 7.03 nmol/L in the O-GlcNAcylated protein; Table S5).

Collectively, these results demonstrate that O-GlcNAcylation at Thr584 significantly enhances PvHMCL’s interaction with bacterial components, resulting in markedly improved antibacterial activity. These findings highlight O-GlcNAcylation as a key post-translational modification regulating hemocyanin-mediated immune defense in *P. vannamei*.

## Discussion

Our study uncovers a previously unrecognized mechanism of immune regulation in crustaceans, demonstrating that bacterial infection activates the HBP in *P. vannamei*, thereby promoting O-GlcNAcylation of hemocyanin (PvHMC). This dynamic post-translational modification (PTM) substantially enhances the antimicrobial properties of PvHMC, identifying O-GlcNAcylation as a critical regulatory node linking glucose metabolism to innate immune function in shrimp.

These findings align with the broader framework of immunometabolism, in which pathogen recognition triggers remodeling to support immune defense. In vertebrates, Toll-like receptor signaling and microbial exposure enhance glucose uptake and reprogram central carbon metabolism, including glycolysis, the pentose phosphate pathway (PPP), and the HBP (6, 37, 38). Comparable pathogen-induced metabolic adaptations have been reported in invertebrates, including other crustaceans (39), underscoring the evolutionary conservation of metabolic-immune coupling. In penaeid shrimp, glucose metabolism is particularly important for resistance to bacterial pathogens such as *V. parahaemolyticus* and *V. alginolyticus*, which deploy virulence strategies that manipulate host glucose metabolic pathways to evade immune responses (18).

Consistent with this paradigm, we show that *V. parahaemolyticus* infection upregulates the major glucose transporter PvGLUT1, likely increasing glucose flux not only through glycolysis but also into the PPP and HBP (14, 40). Transcriptome analyses further revealed substantial upregulation of key HBP components, including *PvGFAT1*, *PvUAP*, and *PvOGT*, following challenge with *Vibrio parahaedmonicus* and *S. iniae* (Fig. 1). Enhanced HBP flux is well known in mammalian systems to evelate protein O-GlcNAcylation as a mechanism to fine-tune immune signaling and effector functions (38, 41). Supporting this model, gene or pharmacological suppression of PvGLUT1, PvGFAT1, or PvOGT reduced both global and PvHMC-specific O-GlcNAcylation, whereas inhibition of PvOGA produced the opposite effect (Fig. 2). Moreover, disruption of this glucose uptake–HBP–O-GlcNAc cycling axis attenuated infection-induced increases in O-GlcNAcylation (Fig. 3), indicating a tight functional link between metabolic reprogramming and activation of antibacterial effector mechanisms.

Central to this regulatory axis are PvOGT and PvOGA, enzymes that are highly conserved from nematodes to mammals (42, 43). We demonstrate for the first time in shrimp that PvOGT directly associates with and O-GlcNAcylates PvHMC, while PvOGA reverses this modification (Fig. 4). Given that enzyme-substrate interactions involving PTMs are typically transient (44), we propose that bacterial infection promotes both increased PvOGT-PvHMC co-localization and elevated local UDP-GlcNAc availability, thereby facilitating efficient modification and enabling detection of their interaction by co-immunoprecipitation. Notably, pathogenic bacteria (*V. parahaemolyticus* and *S. iniae*), but not probiotic species, enhanced PvOGT-PvHMC association and hemocyanin O-GlcNAcylation, indicating selective engagement of this pathway during harmful infections, particularly those caused by Gram-negative *Vibrio* species.

While previous studies have established that N- and O-glycosylation modulate hemocyanin function in mollusks and crustaceans (31, 45, 46, 47, 48), our data provide the first direct evidence that O-GlcNAcylation enhances hemocyanin’s antibacterial and agglutinating activities in shrimp (Fig. 5). Recent work from our group showed that mannosylation, a form of N-glycosylation, promotes host defense *via* mannose receptor-mediated endocytosis (48). However, O-GlcNAcylation is fundamentally distinct from mannosylation in both structure and function. Whereas mannosylation generates complex, branched glycan structures (49), O-GlcNAcylation involves the reversible addition of a single GlcNAc moiety to serine or threonine residues (9). This structural form likely underlies a functional division of labor: O-GlcNAcylated hemocyanin mediates rapid pathogen recognition and neutralization, while mannosylated hemocyanin contributes to subsequent cellular clearance mechanisms. Such temporal stratification mirrors mammalian systems, where O-GlcNAcylation enhances rapid antiviral and inflammatory responses, including regulation of SAMHD1, MAVS signaling, and STAT3 activity (15, 50). The infection-induced elevation of PvHMC O-GlcNAcylation therefore reflects a conserved strategy whereby dynamic PTMs fine-tune immune effector functions across metazoans, and even in prokaryotic contexts (51, 52),

O-GlcNAcylation frequently participates in regulatory crosstalk with phosphorylation at overlapping or adjacent residues (53). Our site-specific analyses revealed that the large hemocyanin subunit (PvHMCL) harbors more O-GlcNAcylation sites than the small subunit (PvHMCS), with four residues (Ser70, Ser191, Ser192, and Thr584) identified as dual sites for O-GlcNAcylation and phosphorylation (Fig. 6). Among these, Thr584 emerged as a critical regulatory residue. Pathogen-induced O-GlcNAcylation at Thr584 was essential for PvHMC's antibacterial activity, as substitution of this residue with alanine abolished the functional enhancement conferred by O-GlcNAcylation, whereas modification of the wild-type protein preserved and augmented antimicrobial capacity (Fig. 7).

These findings integrate with prior work demonstrating that hemocyanin's activity is regulated by multiple PTMs. Dephosphorylation of Thr517 by PP2A enhances bacterial agglutination (29), HDAC3-mediated deacetylation of Lys481/Lys484 promotes LPS binding and pathogen clearance (30), and ubiquitination by PvMULAN influences hemocyanin stability and complex assembly (54). By identifying site-specific O-GlcNAcylation at Thr584 as a direct enhancer of hemocyanin-mediated immunity, our study adds a new regulatory layer to the PTM network governing crustacean immune defense. The dual modification of Thr584 by O-GlcNAcylation and phosphorylation is consistent with a 'Yin-Yang' regulatory paradigm, a mechanism widely implicated in mammalian immune regulation (55). Our data suggest that O-GlcNAcylation at Thr584 may function as a primary regulatory switch, coordinating additional PTMs to precisely modulate hemocyanin activity during infection.

At the mechanistic level, PTMs such as O-GlcNAcylation regulate protein function by altering enzymatic activity, intermolecular interactions, conformational stability, and complex assembly (38, 56). We show that O-GlcNAcylation of PvHMCL at Thr584 significantly enhances its affinity for bacterial components, including LPS and PGN (Fig. 7). Molecular docking analyses indicate that O-glycosylation stabilizes a high-affinity binding conformation through additional hydrogen bonding, resulting in an approximately 24-fold reduction in the dissociation constant for LPS. This structural stabilization likely underlies the enhanced capacity of O-glycosylated hemocyanin to bind and neutralize Gram-negative pathogens such as *V. parahaemolyticus, V. alginolyticus,* and *P. damselae*. These findings are consistent with observations in mollusks, including *Helix lucorum* and *Rapana venosa*, where hemocyanin glycosylation augments antiviral activity through carbohydrate-mediated interactions (47). Conversely, deglycosylation of hemocyanins from multiple species such as *Rapana thomasiana*, *Concholepas concholepas*, *Fissurella latimarginata*, and *Megathura crenulata* disrupts oligomeric structure and compromises biological activity (31, 57, 58). Together, these studies underscore that glycosylation, including O-GlcNAcylation, is a dynamic and essential regulator, rather than a passive structural feature, of hemocyanin-mediated immune defense.

**Figure 8:**
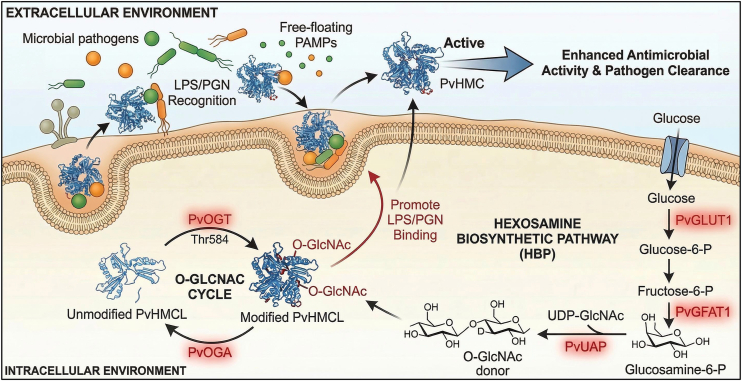
HBP enhances the antibacterial activity of *Pv*HMC through OGT-mediated O-GlcNAcylation of *Pv*HMCL at Thr584.

In summary, this work establishes hemocyanin O-GlcNAcylation as a central regulatory mechanism in the antibacterial immunity of *P. vannamei*. We demonstrate that pathogen-induced metabolic reprogramming enhances HBP activity and promotes both global and hemocyanin-specific O-GlcNAcylation, with PvOGT-mediated modification at Thr584 of PvHMCL driving conformational changes that increase PAMP binding and markedly improve bacterial agglutination, killing, and clearance (Fig. 8). These findings position O-GlcNAcylation as an integral component of metabolic-immune crosstalk in crustaceans and highlight the evolutionary significance of glycosylation in shaping innate immunity. Beyond advancing fundamental understanding, this work provides a conceptual framework for applied strategies in shrimp aquaculture, including the development of O-GlcNAc-based biomarkers for early infection detection and glycosylation-targeted immunostimulants, such as OGT activators or OGA inhibitors (*e.g.*, Thiamet-G), to enhance disease resistance and sustainability.

## Experimental procedures

### Experimental animals

*P. vannamei* (5–8 g) were obtained from Shantou Huaxun Aquatic Product Corporation and maintained in recirculating artificial seawater tanks (salinity: 5 ppt; temperature: 25 °C) for 3 days prior to experimentation. During acclimation, shrimp were fed a commercial diet containing 35% protein once daily. All animal experiments were conducted in accordance with the guidelines and ethical approval of the Animal Research and Ethics Committees of Shantou University.

### Cell and bacteria cultures

*Drosophila* S2 cells (kindly provided by Dr Haoyang Li, School of Marine Science, Sun Yat-sen University) were cultured at 27 °C in Schneider’s *Drosophila* Medium (Invitrogen, 21720024) supplemented with 10% fetal bovine serum (FBS) (Thermo Fisher Scientific, A3160901). Human embryonic kidney (HEK293T) cells (ATCC CRL-1573) were cultured at 37 °C with 5% CO_2_ in Dulbecco’s Modified Eagle Medium (DMEM) (Thermo Fisher Scientific, C11995500BT), also supplemented with 10% FBS. S2 cells were tested for *mycoplasma* contamination using the MycoAlert *Mycoplasma* Detection Kit (Macklin, China, O931116-25 T/EA). HEK293T cells were authenticated upon receipt by PCR-based STR profiling (IGE Bio) and tested for *mycoplasma* using the One-step *Mycoplasma* Detection Kit. Both cell lines were confirmed negative for *mycoplasma* before experiments.

Bacterial strains, including *Escherichia coli* BL21 and DH5α (TransGen Biotech), *V*. *parahaemolyticus* (MCCC1H00057, GenBank: FJ161313.1), *V*. *alginolyticus* (MCCC1A11092, GenBank: EU155488.1), *P*. *damselae* (MCCC1H00017, GenBank: NR_040831.1), *S. iniae* (ATCC29178, GenBank: NZ_JH930418.1), *S*. *aureus* (MCCC1A02584, GenBank: AP017922.1), *E*. *faecalis* (CICC20440, GenBank: CP046022), *P*. *pentosaceus* (CICC22134, GenBank: CP046938.1), and *B. subtilis* (CICC20445, GenBank: MT125883), were obtained from our laboratory collections. All strains were cultured in Luria-Bertani (LB) medium at 37 °C for 16 h, diluted 1:100 in fresh medium, and harvested at the exponential growth phase (OD_600_ = 0.6) for downstream applications.

### Tissue collection, protein preparation, and western blot analyses

Hemolymph was collected from five randomly selected, acclimatized shrimp per group and processed as previously described (30) to separate hemocytes and plasma. Hemocytes were lysed in Western/IP lysis buffer (Beyotime, P0013J) supplemented with protease inhibitors (MedChemExpress, HY-K0010) and clarified by centrifugation at 20,000*g* for 15 min at 4 °C. The resulting supernatants were collected for downstream analyses.

Plasma samples from each group were subjected to size-exclusion chromatography using an ÄKTA Pure system (GE Healthcare; 29018224) equipped with a Superose 6 Increase 10/300 Gl column (GE Healthcare; 29091596) to isolate PvHMC-enriched fractions. The column was equilibrated in Tris–HCl buffer (0.05 M, pH 7.4). For each run, 2 ml of *P. vannamei* plasma was loaded onto the column and eluted at a flow rate of 0.2 ml/min with continuous monitoring of absorbance at 280 nm. Fractions (2 ml each) were collected sequentially. Based on the elution profile, supported by preliminary SDS-PAGE and Western blot analyses, fractions corresponding to the major PvHMC-containing peak were identified and pooled using an identical elution window across all sample types. The pooled fractions were then filtered through a 0.22 μm Millex-GP membrane (Millipore; SLGPR33RB) and either used immediately or stored at −80 °C until further analysis.

For extraction of plasma-associated bacterial proteins, plasma samples were centrifuged at 12,000*g* for 15 min at 4 °C to pellet bacteria. The bacterial pellets were lysed in lysis buffer containing protease inhibitors and clarified by centrifugation at 20,000*g* for 15 min at 4 °C, after which the supernatants were collected.

Protein samples from hemocytes, plasma, and bacteria were mixed with 5 × SDS sample buffer (42 mM Tris-HCl, 10% glycerol, 2.3% SDS, 5% β-mercaptoethanol, 0.002% bromophenol blue, pH 6.8) and heated at 100 °C for 10 min. Proteins were separated by 10% SDS-PAGE and transferred onto PVDF membranes (Millipore, IPVH00010). Western blot was performed using the antibodies listed in the Antibodies section, and signals were detected using an Amersham Imager 600 system (GE Healthcare, 29083463) according to the manufacturer’s instructions.

### PNGase F pretreatment

Enzymatic removal of N-linked glycans was performed according to the manufacturer’s instructions using biotinylated PNGase F (Beyotime; P2330L). Cell lysates or purified PvHMC (100 μg) were denatured in 10 × Glycoprotein Denaturing Buffer at 100 °C for 10 min and cooled to room temperature. Samples were then adjusted to a final reaction volume of 20 μl by addition of 10 × GlycoBuffer 2, 10% NP-40, and nuclease-free H_2_O. Biotinylated PNGase F (1 μl) was added, and reactions were incubated at 37 °C for 4 h.

To remove the biotinylated enzyme, streptavidin magnetic beads (15 μl, resuspended in 1 × Reaction Buffer) were added and incubated at room temperature for 10 min. Beads were magnetically separated, and the supernatants containing deglycosylated proteins were collected. PNGase F-treated samples were subsequently used for Western blot analysis, CTD110.6 immunoprecipitation followed by silver staining, and mass spectrometry.

### Pathogen challenge experiments

Shrimp acclimated to laboratory conditions for at least 3 days were randomly divided into three groups (n = 100 shrimp per group). Individuals were intramuscularly injected with 100 μl of *V. parahaemolyticus* (5.0 × 10^6^ CFU/ml), *S*. *iniae* (5.0 × 10^6^ CFU/ml), or an equal volume of sterile 0.01 M phosphate-buffered saline (PBS; 135 mM NaCl, 2.7 mM KCl, 1.5 mM KH_2_PO_4_, 8 mM K_2_HPO_4_, pH 7.4) as a control. Hemolymph samples were collected at 0, 6, 12, 24, 48, 72, and 96 h post-injection (hpi) for downstream analyses.

### Genomic DNA and total RNA extraction, cDNA synthesis, and real-time quantitative PCR

Genomic DNA (gDNA) and total RNA were extracted from shrimp hepatopancreas, hemocytes, and bacterial cultures using the TIANamp Marine Animals DNA Kit (TIANGEN, DP324) and TRIzol Plus RNA Purification Kit (Invitrogen, 12183555), respectively, following the manufacturers’ protocols. Nucleic acid concentrations were determined with a NanoDrop 2000 spectrophotometer (Thermo Fisher Scientific), and RNA quality was confirmed by A260/280 ratios ≥2.0 and agarose gel electrophoresis.

First-strand cDNA was synthesized using 1.0 μg of RNA with the One-Step gDNA Removal and cDNA Synthesis SuperMix Kit (TransGen Biotech, AT311–02). Gene-specific primers for *Pvglut1* (GenBank: KJ701599.1), *Pvgfat1* (GenBank: KP119675), *Pvuap* (GenBank: XM_027376979.1), *Pvogt* (GenBank: MH797025.1), *Pvoga* (GenBank: MH797026.1), and the reference gene *Pvef1α* (GenBank: GU136229) were designed using Primer Premier 5 (see Table S1). Primers targeting *Vibrio*, *V. parahaemolyticus*, and *S. iniae* were adopted from published studies (59, 60). Quantitative PCR (qPCR) was performed using the qTOWER3G system (Analytik Jena) with thermal cycling conditions: 95 °C for 10 min, followed by 45 cycles of 95 °C for 15 s and 60 °C for 30 s. Relative gene expression was calculated using the 2^−ΔΔCT^ method (61), normalized to *Pvef1α*.

### Cell transfections

*Drosophila* S2 cells (2 × 10^6^ cells/well) were seeded into six-well plates and transfected 12 h later with plasmids pIZ-*Pv*HMCL-Flag, pIZ-*Pv*HMCL-T584A-Flag, or pIZ-*Pv*OGT-V5 using FuGENE HD Transfection Reagent (Promega, E2311), following the manufacturer’s instructions. At 36 h post-transfection, cells were stimulated with LPS (1 μg/ml) or PGN (6 μg/ml) for 3 h. Finally, cells were harvested and split into two aliquots for Western blot and co-immunoprecipitation assays.

### Extraction of bacteria OMPs

OMPs from *V*. *parahaemolyticus* were extracted using a modified sodium lauryl sarcosine (SLS) protocol. Briefly, bacteria were cultured to mid-log phase (OD_600_ = 0.8), pelleted, and washed with 50 mM Tris-HCl (pH 7.4). Cells were resuspended in lysis buffer (50 mM Tris-HCl, 150 mM NaCl, pH 7.4) and lysed on ice by ultrasonication (Scientz, Zhejiang, JY92-II). Lysates were centrifuged at 12,000*g* for 15 min, and the supernatants were ultracentrifuged at 100,000*g* for 1 h at 4 °C. Next, pellets containing membrane fractions were resuspended in 2% SLS in 50 mM Tris-HCl (pH 7.5) and incubated at room temperature for 40 min. After ultracentrifugation (100,000*g*, 1 h, 4 °C), the resulting OMP pellets were solubilized in lysis buffer containing 1% Triton X-100 and stored at −40 °C until use.

### Plasmid constructs, protein expression, and purification

The coding sequences for the functional domains of *P*. *vannamei* O-GlcNAc transferase (*PvOGT*, GenBank: MH797025.1) and O-GlcNAcase (*PvOGA*, GenBank: MH797026.1) were cloned into the pGEX-6P-1 vector (GE Healthcare, 28-9546-48) at the BamHI site using the ClonExpress II One Step Cloning Kit (Vazyme, Nanjing, China, C112). The resulting constructs, designated *Pv*OGT-F-GST and *Pv*OGA-F-GST, were transformed into *E*. *coli* BL21 cells for protein expression. Cultures were induced with 1 mM IPTG (GenStar, B117) at 16 °C for 12 h, and recombinant proteins were purified using Glutathione-Sepharose 4B beads (GE Healthcare, 17075601).

Plasmids expressing the wild-type large subunit of *P. vannamei* hemocyanin (*Pv*HMCL-WT-Flag) were constructed as previously described (30). Site-directed mutants targeting putative O-GlcNAcylation sites (S70A, S191A, S192A, T584A) were generated using the Fast Mutagenesis System Kit (TransGen Biotech, FM201-01) and cloned into the pcDNA3.1+ vector (Thermo Fisher Scientific) at the XhoI site. All constructs were transformed into *E. coli* DH5α, purified, and transfected into HEK293T cells using Lipofectamine 2000 (Thermo Fisher Scientific, 11668030). After 48 h, cells were lysed in RIPA buffer (Beyotime, P0013B) and *Pv*HMCL-Flag-tagged proteins were purified using Anti-Flag M2 Magnetic Beads (MedChemExpress, Monmouth Junction, HY-K0207), followed by elution with 3×Flag peptide. The purified proteins were concentrated using Amicon Ultra-0.5 filters (Millipore, UFC5010BK) and verified by SDS-PAGE.

For insect cell expression, *PvOGT*, *PvHMCL-WT*, and *PvHMCL-T584A* were cloned into the pIZ/V5-His vector (Invitrogen, V8000-01) at BamHI/XhoI sites, generating pIZ-*Pv*OGT-V5/His, pIZ-*Pv*HMCL-Flag, and pIZ-*Pv*HMCL-T584A-Flag constructs. All primer sequences are listed in Table S1.

### Antibodies

The following primary antibodies were used for Western blot: mouse anti-O-GlcNAc (sc-59623; Santa Cruz Biotechnology, 1:300), rabbit anti-phospho-Ser/Thr (ab9344; Abcam, Cambridge, UK, 1:500), mouse anti-GLUT1 (sc-377228; Santa Cruz Biotechnology, 1:300), mouse anti-GFAT1 (sc-377479; Santa Cruz Biotechnolog, 1:300), mouse anti-OGT (sc-74546; Santa Cruz Biotechnology CA, 1:250), mouse anti-OGA (sc-376429; Santa Cruz Biotechnology, 1:250), mouse anti-Tubulin (T6074; Sigma-Aldrich, St Louis, MO, USA, 1:3000), mouse anti-GST (HT601–01; TransGen Biotech, Beijing, China, 1:3000), rabbit anti-V5 (V8137; Sigma-Aldrich, St Louis, MO, USA, 1:1000), mouse anti-Flag (F18044; Sigma-Aldrich, 1:3000), and custom rabbit anti-*P. vannamei* hemocyanin antisera (62) at 1:5000. For immunocytochemistry, mouse anti-OGT (1:50) and rabbit anti-*Pv*HMC (1:200) were used, while for ELISA, rabbit anti-*Pv*HMC was applied at 1:1000.

The secondary antibodies included goat anti-rabbit IgG-HRP (Thermo Fisher Scientific, Waltham, MA, USA, 31,460) and goat anti-mouse IgG-HRP (Thermo Fisher Scientific, 31430), both used at 1:5000 for Western blot. For ELISA, peroxidase-conjugated goat anti-rabbit IgG (1:2000) was used. In the immunocytochemistry assay, peroxidase-conjugated goat anti-mouse IgG (1:100) and peroxidase-conjugated goat anti-rabbit IgG (1:500) were employed.

### RNA interference and inhibitor treatment

Small interfering RNAs (siRNAs) targeting *PvGLUT1* (GenBank: KJ701599.1), *PvGFAT1* (GenBank: KP119675), *PvOGT* (GenBank: MH797025.1), *PvOGA* (GenBank: MH797026.1), and a scrambled negative control (siNon) were designed (Table S1) and synthesized by GenePharma. Pre-acclimatized shrimp were randomly assigned to three groups (n = 20 per group) and injected with 100 μl (0.5 μg/g body weight) of siRNA or siNon.

For chemical inhibition, shrimp (n = 10 per group) received injections of one of the following inhibitors: WZB117 (100 μg/ml; GLUT1 inhibitor; MedChemExpress, HY-19331), DON (500 μg/ml; GFAT inhibitor; MedChemExpress, HY-108357), OSMI4 (500 μg/ml; OGT inhibitor; MedChemExpress, HY-114361), or Thiamet G (100 μg/ml; OGA inhibitor; MedChemExpress, HY-12588). Control shrimp were injected with sterile PBS (0.01 M, pH 7.4). Hemolymph was collected from five shrimp per group at various time points for hemocyte and plasma isolation, followed by analysis of target gene expression and *Pv*HMC O-GlcNAcylation.

### Pull-down, Co-immunoprecipitation, and immunoprecipitation coupled with mass spectrometry

For GST pull-down assays, purified hemocyanin was incubated with GST-tagged *Pv*OGT-F, *Pv*OGA-F, or GST alone at 4 °C for 30 min. After extensive washing with PBS containing 0.5% Triton X-100, bound proteins were analyzed by SDS-PAGE followed by Western blot as described above.

Co-immunoprecipitation (Co-IP) assays were performed according to established protocols (23). Briefly, hemocytes and plasma-derived bacterial fractions were washed with anticoagulant buffer and lysed in IP lysis buffer supplemented with protease inhibitors. Lysates were clarified by centrifugation at 20,000*g* for 15 min at 4 °C. Protein concentrations were determined using a BCA Protein Assay Kit (GenStar, E162), adjusted to 1 mg/ml, and pre-cleared. Aliquots of lysate (600 μl) were incubated overnight at 4 °C with 2 μg of the indicated antibodies, followed by incubation with Protein A/G Magnetic Beads (MedChemExpress, HY-K0202) for 1 h. Immunocomplexes were washed, eluted by boiling in SDS sample buffer, and analyzed by SDS-PAGE and Western blot.

For O-GlcNAc proteome analysis, hemolymph proteins were subjected to immunoprecipitation was performed using anti-O-GlcNAc antibodies following PNGase F pretreatment to remove N-linked glycans. Protein L (MedChemExpress, HY-K0205) agarose beads were used to capture immune complexes. Eluted proteins were resolved by SDS-PAGE and visualized by Coomassie Brilliant Blue staining. Distinct gel bands were excised and processed for mass spectrometry (Wininnovate Bio) as previously described (15). Briefly, gel pieces were destained, digested with Trypsin Gold (Promega, V5280), and the resulting peptides were analyzed using an Easy nLC 1200 system coupled to a Q Exactive mass spectrometer (Thermo Fisher Scientific).

MS/MS data were processed using PEAKS Studio 8.5 (AB SCIEX) and searched against the *P. vannamei* UniProtKB database (https://www.uniprot.org). Variable modifications included methionine oxidation, N-terminal acetylation, deamidation (NQ), pyroglutamate formation from E/Q, with carbamidomethylation of cysteine specified as a fixed modification. Precursor and fragment mass tolerances were set to 10 ppm and 0.05 Da, respectively. Proteins were ranked based on abundance changes in CTD110.6 immunoprecipitates from *V. parahaemolyticus*–challenged samples relative to PBS controls, and candidate proteins were defined using a fold-change threshold (|log2 ratio| ≥ 1).

### *In vitro* O-GlcNAcylation and O-GlcNAc cleavage assay

*In vitro* O-GlcNAcylation of purified *Pv*HMC or *Pv*HMCL proteins was performed as previously described (63). Briefly, 1 mg of recombinant GST-PvOGT-F protein was incubated with 2 mg of purified *Pv*HMC or *Pv*HMCL in 2 ml of reaction buffer containing 50 mM Tris-HCl (pH 7.5), 12.5 mM MgCl_2_, 2 mM UDP-GlcNAc (Sigma, U4375), and 1 mM DTT for 4 h at 37 °C. After incubation, samples were separated by SDS-PAGE and analyzed by Western blot using anti-O-GlcNAc antibodies.

For O-GlcNAc removal, 1 mg of O-GlcNAcylated *Pv*HMC or *Pv*HMCL was incubated with 1 mg of recombinant GST-*Pv*OGA-F protein in 500 μl of cleavage buffer (50 mM Tris-HCl, 12.5 mM MgCl_2_, 1 mM DTT, pH 7.5) at 37 °C for 2 h. The reaction products were subjected to immunoblotting with anti-O-GlcNAc antibodies.

### Immunocytochemistry assay

Shrimp hemocytes (3 × 10^5^ cells) were seeded onto confocal dishes (NEST, Shanghai, China, 801002) and fixed with 4% paraformaldehyde for 15 min. Cells were permeabilized with saponin (Beyotime, P0095) for 10 min and washed three times with PBS. After blocking with QuickBlock solution (Beyotime, P0256) for 30 min at room temperature, cells were incubated overnight at 4 °C with primary antibodies (anti-OGT or anti-*Pv*HMC). Following five PBS washes, cells were incubated for 1 h at room temperature with Alexa Fluor 555-conjugated donkey anti-rabbit IgG (Beyotime, A0453) or Alexa Fluor 488-conjugated goat anti-mouse IgG (Beyotime, A0428). Nuclei were counterstained with Hoechst 33342 (Thermo Fisher, 62249) for 5 min. Images were captured using a confocal microscope (Zeiss LSM 880, Oberkochen, 880-201).

### *In vitro* antibacterial and bacteria-binding assays

The antibacterial activity of *Pv*HMC and *Pv*HMCL was assessed by bacterial growth inhibition and agglutination assays, as previously described (64). For the inhibition assay, 50 μl of *Pv*HMC or *Pv*HMCL (100 μg/ml in TBS) was incubated with 50 μl of bacterial suspensions (1 × 10^4^ CFU/ml) of *V. parahaemolyticus*, *V. alginolyticus*, *P. damselae*, *S. iniae*, or *S. aureus* at 37 °C for 2 h. Aliquots (30 μl) were plated on LB agar, incubated at 37 °C for 12 h, and bacterial colony counts were used to calculate inhibition rates as follows:

$$Inhibition\;\left(\%\right)=\frac{colonies\;in\;control-colonies\;in\;sample}{colonies\;in\;control}\times 100\%$$

For bacterial agglutination assays (45), bacterial suspensions (10^8^ CFU/ml) were prepared in TBS-Ca^2+^ buffer (0.05 M Tris, 0.75% NaCl, 0.05 M CaCl_2_) and mixed 1:1 with serial dilutions of *Pv*HMC or *Pv*HMCL. After 30 min at 37 °C incubation, agglutination was observed by light microscopy (Zeiss, Oberkochen, Baden, Germany) and scored qualitatively. The agglutination titer was defined as the highest dilution showing positive agglutination.

For bacterial binding assays (65), 200 μl of *Pv*HMC or TBS was incubated with 2 × 10^8^ CFU/ml of bacterial cells (including *V. parahaemolyticus*, *V. alginolyticus*, *P. damselae*, *S. iniae*, *S. aureus*, *E. faecalis*, *P. pentosaceus*, and *B. subtilis*) at 37 °C for 30 min. Bacteria were collected by centrifugation, washed extensively with TBS, and analyzed by SDS-PAGE and Western blot using anti-*Pv*HMC antibody. All experiments were performed in triplicate and independently repeated at least three times.

### ELISA assay

The interaction between O-GlcNAcylated hemocyanin and bacterial components, including LPS, PGN, or OMPs, was assessed using an enzyme-linked immunosorbent assay (ELISA). Briefly, 100 μl of varying concentrations of LPS (0, 25, 50, or 100 μg/well; Sigma, L2880), PGN (0, 25, 50, or 100 μg/well; Sigma, St Louis, 77140), or OMPs (0, 100, 200, or 400 ng/well; prepared as described above) were coated onto 96-well ELISA plates and incubated overnight at 4 °C. After blocking with 200 μl of 3% BSA in PBS (0.15 M, pH 7.4) for 2 h at room temperature, plates were washed four times with PBS. Purified *Pv*HMC (50 μg/ml in PBS with 0.1 mg/ml BSA) was added (100 μl/well) and incubated for 3 h at room temperature. Wells were washed again and incubated for 1 h with primary antibodies, followed by incubation with peroxidase-conjugated secondary antibodies for 45 min. Color development was achieved using 3,3′,5,5′-tetramethylbenzidine (Beyotime, P0209), and the reaction was stopped with 2 M H_2_SO_4_. Absorbance was measured at 450 nm using a microplate reader (Tecan Spark). All treatments were performed in triplicate, and experiments were independently repeated at least three times.

### Mapping of O-GlcNAcylation sites

Mass spectrometry (MS) was used to identify O-GlcNAcylation sites on *P. vannamei* hemocyanin, following previously described protocols (14). Briefly, O-GlcNAc-enriched hemocyanin from shrimp plasma collected 48 h after *V*. *parahaemolyticus* challenge was separated by SDS-PAGE. Differential protein bands were excised, reduced with DTT, alkylated with iodoacetamide, and digested with trypsin overnight. Peptides were analyzed using an Easy-nLC 1000 system coupled to an Orbitrap Exploris 480 mass spectrometer (Thermo Fisher Scientific) in data-dependent acquisition mode. The 30 most intense precursors were selected for higher-energy collision dissociation (HCD). Raw MS data were processed using Proteome Discoverer 2.0 (Thermo Fisher Scientific) and searched using the Sequest algorithm against the *P. vannamei* UniProtKB database (https://www.uniprot.org/). Search parameters included a precursor mass tolerance of 10 ppm, fragment mass tolerance of 0.6 Da, allowance for two missed cleavages, fixed carbamidomethylation of cysteine, variable oxidation of methionine, phosphorylation of serine/threonine/tyrosine, and O-GlcNAc (HexNAc [+203.0794 Da]) on serine/threonine. Peptides were filtered using a false discovery rate (FDR) threshold of 5% *via* the Percolator node.

*In silico* prediction of potential O-GlcNAcylation sites in *Pv*HMC was performed using O-GlcNAcPRED-DL (https://www.oglcnac.org/pred_dl/), NetOGlyc-4.0 (https://services.healthtech.dtu.dk/services/NetOGlyc-4.0/), and YinOYang 1.2 (https://services.healthtech.dtu.dk/services/YinOYang-1.2/). Amino acid sequences of *Pv*HMCS (UniProtKB: A0A1Y0DT76, Q26180) and *Pv*HMCL (UniProtKB: Q9NFY6, G9BYP1, A0A088MK65, A0A059TFW7, A0A059TFW8, A0A059TEX0, A0A059TIR8, A0A059TGC6, A0A059TEW9, A0A423SGT1, and A0A0G2YAK1) were submitted in FASTA format. Prediction results from the three tools were aggregated, visualized as heat maps, and compared to the experimental MS results.

### Homology modeling and molecular docking

The three-dimensional structure of *Pv*HMC was predicted using AlphaFold version 2.3.2 based on the latest Protein Data Bank sequences (66). The predicted models were evaluated using confidence scores (C-scores). To model O-GlcNAc modification at Thr584, UCSF Chimera (67) was used to manually add the glycosylation group, and AMBER14SB force field parameters were applied. Protonation states were assigned using the H++3 online tool (68). Potential ligand-binding sites were identified using SiteMap (69). Molecular structures of LPS and PGN were generated with RDKit, and their atomic charges were assigned using AM1-BCC calculations in UCSF Chimera (70). Molecular docking between *Pv*HMC and bacterial ligands (LPS and PGN) was performed using AutoDock 4.2 with the Lamarckian genetic algorithm. The docking grid was set to 22.5 Å × 22.5 Å × 22.5 Å with a spacing of 0.375 Å, and 100 independent runs were carried out for each ligand-protein pair (71). Binding free energy calculations were performed using the g_mmpbsa module, and interaction diagrams were generated using LigPlot+ (https://www.ebi.ac.uk/thornton-srv/software/LigPlus/). All structural visualizations were prepared using PyMOL (http://www.pymol.org/).

### Statistical analysis

Data are presented as mean ± SEM. Statistical significance was determined using Student’s *t* test or one-way ANOVA with GraphPad Prism 9 and IBM SPSS Statistics (version 20; IBM). *p <* 0.05 was considered statistically significant.

## Data availability

The mass spectrometry proteomics data generated in this study have been deposited to the ProteomeXchange Consortium *via* the PRIDE partner repository under the dataset identifiers PXD073347 (candidate O-GlcNAcylated proteins in shrimp after *V. parahaemolyticus* challenge) and PXD046737 (hemocyanin O-GlcNAcylation site mapping). All other data are contained within the article and its supporting information.

## Supporting information

This article contains supporting information.

## Conflict of interest

The authors declare that they have no conflicts of interest with the contents of this article.
