# Supplementary material for: Hexosamine biosynthesis drives hemocyanin O-GlcNAcylation to potentiate antibacterial immunity in shrimp
Source: J Biol Chem. 2026 May 28;302(7):113213. doi: 10.1016/j.jbc.2026.113213 (PMC13310628; doi:10.1016/j.jbc.2026.113213)
Supplement: Supplementary Materials [file mmc1.docx]

**Supplementary data**


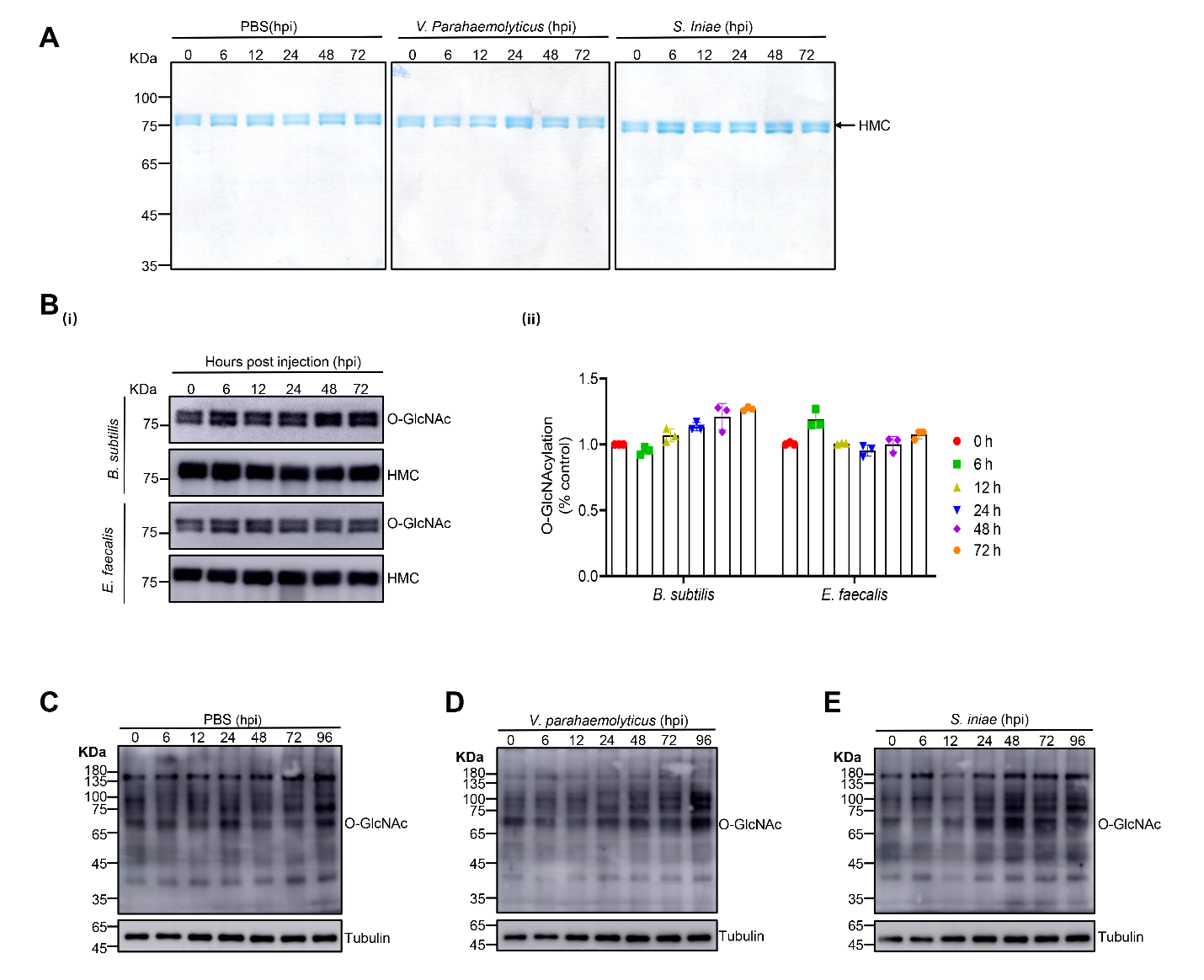


**Figure S1.** **Bacterial infection promotes protein O-GlcNAcylation in *P. vannamei*. (A)** SDS-PAGE and Coomassie Brilliant Blue staining of SEC-purified PvHMC from hemolymph collected at 0, 6, 12, 24, 48, and 72 h after PBS, *V. parahaemolyticus*, or *S. iniae* injection. **(B)** Western blot analysis of O-GlcNAc-modified *Pv*HMC in *Penaeus vannamei* hemolymph after injection with PBS. (i) Western blot analysis and (ii) Relative gray value analysis. **(C-E)** Western blot analysis of hemocytes total protein O-GlcNAcylation level after injection with (C) PBS, (D) *V. parahaemolyticus*, (E) *S. iniae.*


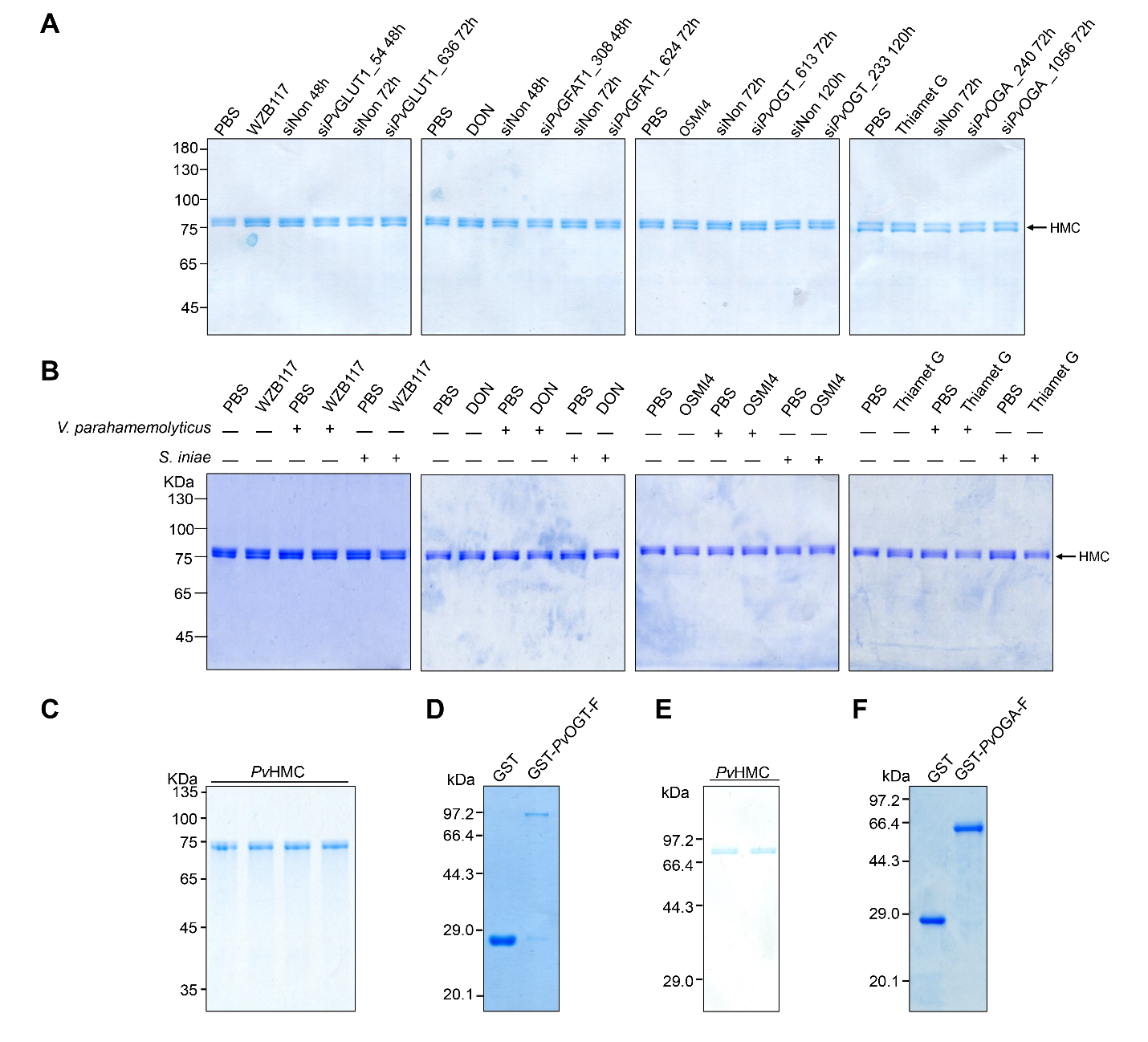


**Figure S2. Coomassie staining of SEC-purified PvHMC and recombinant proteins. (A–B)** SDS–PAGE with Coomassie Brilliant Blue staining of SEC-purified PvHMC to show purification/inputs under the indicated inhibition/knockdown conditions (A), with or without bacterial challenge (B). **(C-D)** Coomassie Brilliant Blue staining showing inputs (PvHMC, GST, GST–PvOGT-F) for the O-GlcNAcylation assay. **(E-F)** Coomassie staining showing inputs (PvHMC, GST, GST–PvOGA-F) for the O-GlcNAc removal assay.


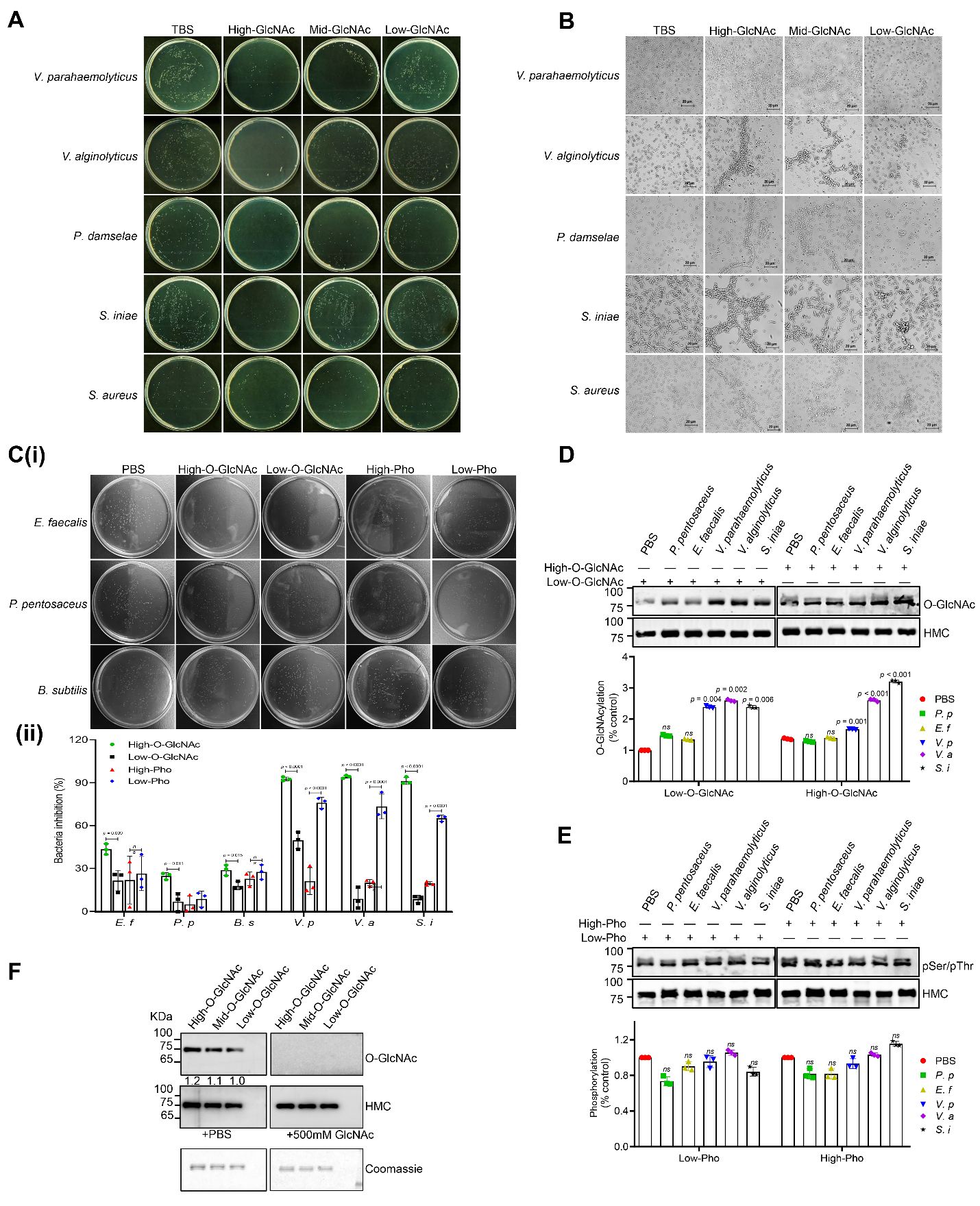


**Figure S3. O-GlcNAcylation of *Pv*HMC affects its antibacterial activity. (A)** Bacterial colonies in petri dishes treated with high-GlcNAc, mid- O-GlcNAc, and low-O-GlcNAc, or sterile 0.01M pH 7.4 TBS as the negative control. **(B)** Microscopic view (400 ×) of the agglutinate formed by high-GlcNAc, mid- O-GlcNAc, and low-O-GlcNAc proteins with bacteria, or TBS (used as negative control). **(C)** Bacterial inhibition of different O-GlcNAcylated *Pv*HMC samples (high, mid, low) and different phosphorylated *Pv*HMC samples (high and low) against *E. faecalis, P. pentosaceus, B. subtilis.* **(D-E)** Western blot analysis elucidates the effects of factors *P. pentosaceus*, *E. faecalis*, *V. parahaemolyticus*, *V. alginolyticus* and *S. iniae* on the post-translational modifications of hemocyanin after *in vitro* incubation. (D) O-GlcNAcylation and (E) phosphorylation. **(F)** Specificity controls for CTD110.6 detection of *Pv*HMC O-GlcNAcylation.


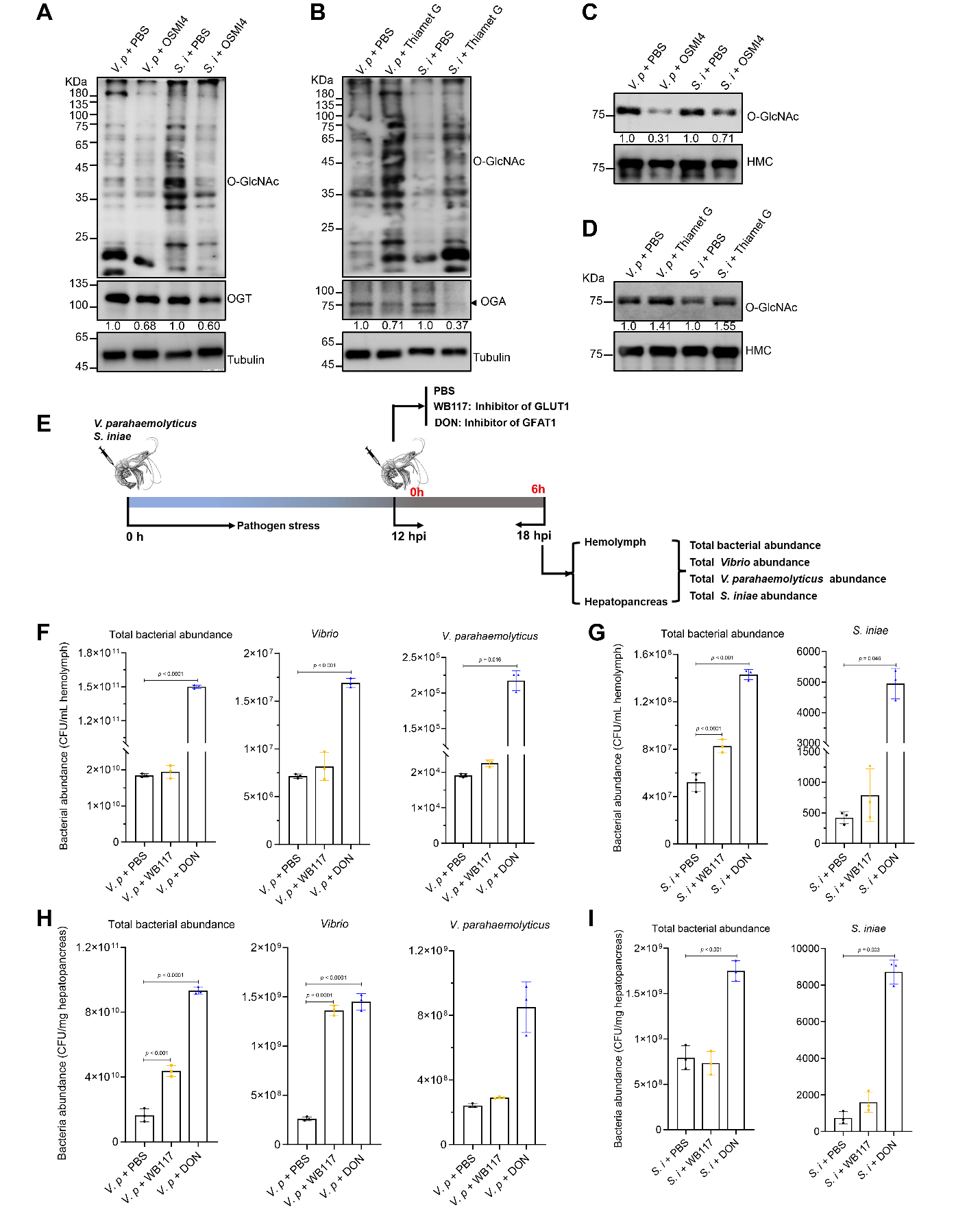


**Figure S4. Inhibition of *Pv*GLUT1 and *Pv*GFAT1 from HBP promotes bacterial replication. (A-D)** Immunoblot analysis of total protein O-GlcNAc in hemocytes (A-B) and PvHMC O-GlcNAcylation in plasma (C-D) after chemical inhibition of PvOGT (A, C) or PvOGA (B, D), followed by challenge with *V. parahaemolyticus* or *S. iniae*. **(E)**. Schematic of shrimp injections and pathogen challenge. **(F-G).** Bacterial abundance in hemolymph after chemical inhibition of *Pv*GLUT1/*Pv*GFAT1 followed by (F) *V. parahaemolyticus* and (G) *S. iniae* infection. **(H-I)** Total bacterial and Vibrio abundance in hepatopancreas post-chemical inhibition followed by infection with (H) *V. parahaemolyticus* and (I) *S. iniae*. Data represent mean ± S.E.M. from three independent experiments. One-way ANOVA was used to assess significance.


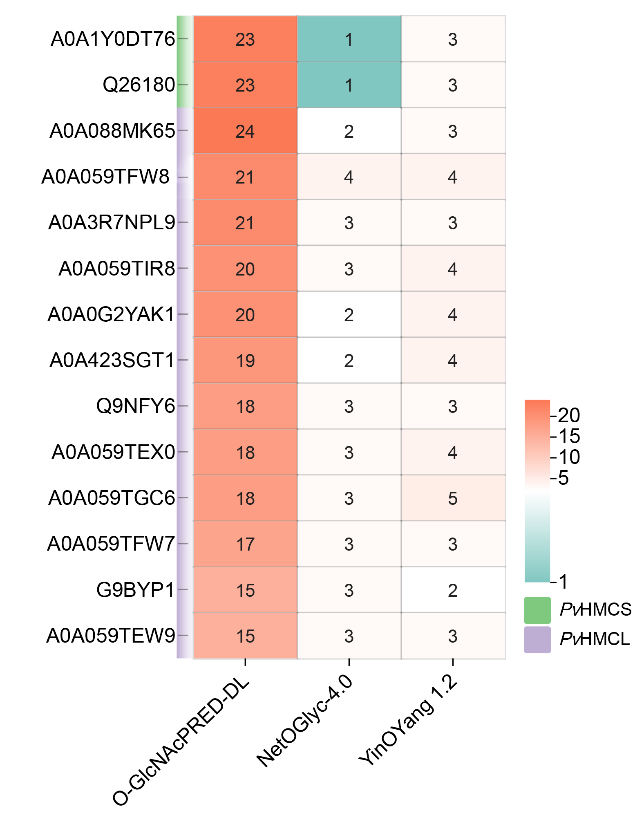


**Figure S5. Predicted O-GlcNAcylation sites of** **different variants of** ***Pv*HMC with their UniProtKB registration numbers**. Red to green indicates high to low prediction scores that represent the number of *Pv*HMC O-GlcNAcylation site corresponding to each prediction website.


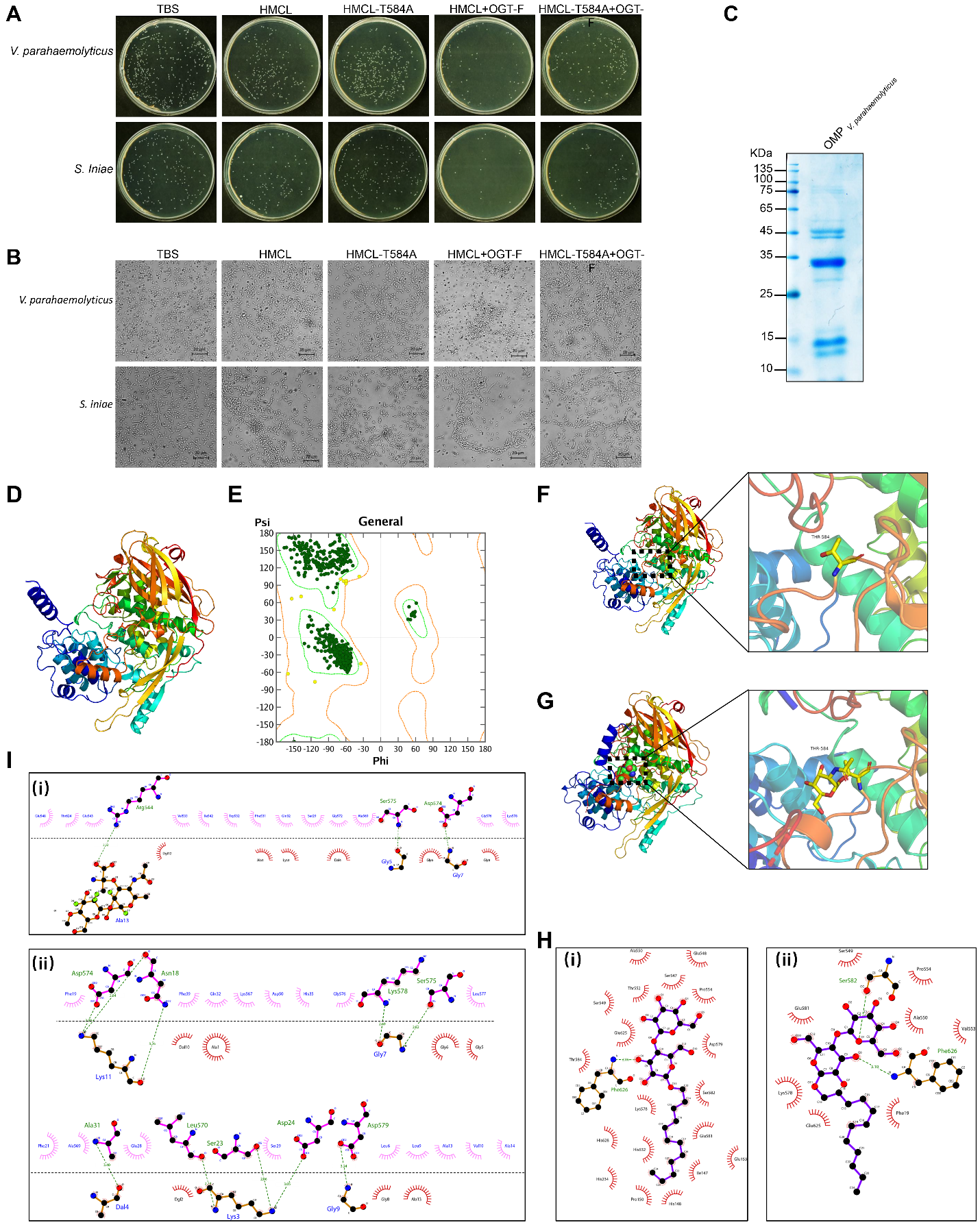


**Figure S6. O-GlcNAcylation of *Pv*HMCLThr584 is closely related to immunity. (A)** Bacterial colonies in Petri dishes treated with *Pv*HMCL, *Pv*HMCL-T584A, *Pv*HMCL+*Pv*OGT-F and *Pv*HMCL-T584A+*Pv*OGT-F (100 μg/mL), or TBS as negative control. **(B)** Microscopic view (400 ×) of the agglutinate formed by *Pv*HMCL, *Pv*HMCL-T584A, *Pv*HMCL+*Pv*OGT-F and *Pv*HMCL-T584A+*Pv*OGT-F (100 μg/mL) with bacteria, TBS was used as negative control. **(C)** SDS-PAGE analysis of the OMPs of *V. parahaemolyticus.* **(D).** The homology model of *Pv*HMCL monomer. **(E)** Ramachandran plot for *Pv*HMCL monomer. The green dots represent favored regions, the yellow dots indicate generally allowed regions, and the others are irrational regions. **(F-G)** The detailed three-dimensional (3D) mode of the wild type *Pv*HMCL (F) and O-GlcNAc-*Pv*HMCL-Thr584 (G). **(H-I)** General overview and local overview 2D of the best-ranked docking pose of LPS (H) or PGN (I) binding with *Pv*HMCL (i) and *Pv*HMCL-GlcNAcT584 (ii). Hydrogen-bonding interactions highlighted by the green dashed line and hydrophobic interactions highlighted by the arc-shaped eyelash pattern in 2D illustrations, in which the protein amino acids are located above the dotted line, while the PGN amino acids and modified glycosides are located below it.

**Table S1**. **Main primers used in this paper**

| **Primer name** | **Sequence (5'-3')** |
| --- | --- |
| **Protein expression** | |
| *Pv*OGT-F-GST-F | CGCGGATCCATGACCTCAGATGGCCGT |
| *Pv*OGT-F-GST-R | TCCCCCGGGTCAACCACTATATTTCTTCC |
| *Pv*OGA-F-GST-F | CGCGGATCCATGAAAAACTTCGTCTGCG |
| *Pv*OGA-F-GST-R | TCCCCCGGGTCAGCCAGTTGGGCAATC |
| *Pv*OGT-V5-F | TTGGTACCGAGCTCGGATCCATGAAGATGACGCAACAATCTA |
| *Pv*OGT-V5-R | GCGGGCCCTCTAGACTCGAGCGCCACTCAGTGATGTGGTCT |
| *Pv*HMCL-Flag-F | CACAGTGGCGGCCGCATGAAGGTCCTGCTGCTCCTCG |
| *Pv*HMCL-Flag-R | GCGGGCCCTCTAGACTTACTTATCGTCGTCATCCTTGTAATCATGATGGATATGCTCGCCA |
| *mut*-*Pv*HMCLS70A-F | ATCCCATTACGCCGATGGAGGT |
| *mut*-*Pv*HMCLS70A-R | ACCTCCATCGGCGTAATGGGAT |
| *mut*-*Pv*HMCLS191A-F | TAAATTTAAGGCTAGTTTCAC |
| *mut*-*Pv*HMCLS191A-R | GTGAAACTAGCCTTAAATTTA |
| *mut*-*Pv*HMCLS192A-F | ATTTAAGTCTGCTTTCACTGGA |
| *mut*-*Pv*HMCLS192A-R | TCCAGTGAAAGCAGACTTAAAT |
| *mut*-*Pv*HMCLT584A-F | CGAGAGTGCCGCTGGTATTCC |
| *mut*-*Pv*HMCLT584A-R | GGAATACCAGCGGCACTCTCG |
| **siRNA** | |
| si*Pv*GLUT1_54-F | GCUUGGAAUGUUCCAGUUUTT |
| si*Pv* GLUT1_54-R | AAACUGGAACAUUCCAAGCTT |
| si*Pv*GLUT1_636-F | GCUCAUGUCACGCCAGUUATT |
| si*Pv*GLUT1_636-R | UAACUGGCGUGACAUGAGCTT |
| si*Pv*GFAT_308-F | GGGAUGAUGUGUGCUUCUUTT |
| si*Pv*GFAT_308-R | AAGAAGCACACAUCAUCCCTT |
| si*Pv*GFAT_624-F | GGAGGUGUUGAGUCUGGAUTT |
| si*Pv*GFAT_624-R | UAACUGGCGUGACAUGAGCTT |
| si*Pv*OGT_613-F | GCAGUGCUACUGCUGCUUUTT |
| si*Pv*OGT_613-R | AAAGCAGCAGUAGCACUGCTT |
| si*Pv*OGT_233-F | GCAUCAACCAGGAUGGCAUTT |
| si*Pv*OGT_233-R | AUGCCAUCCUGGUUGAUGCTT |
| si*Pv*OGA_240-F | GCACCUUACAGCCCUCAUUTT |
| si*Pv*OGA_240-R | AAUGAGGGCUGUAAGGUGCTT |
| si*Pv*OGA_1056-F | CCGUCCCAUUUCUGCAUUUTT |
| si*Pv*OGA_1056-R | AAAUGCAGAAAUGGGACGGTT |
| siNon-F | UUCUCCGAACGUGUCACGUTT |
| siNon-R | ACGUGACACGUUCGGAGAATT |
| **Real-time PCR** |  |
| *Pv*hmc-qF | ATTGAACGCAAGTCCACG |
| *Pv*hmc-qR | CTGAGTCGGCATCACCAT |
| *Pv*glut1-qF | CGTGCATCAAGTCATGTTG |
| *Pv*glut1-qR | CATGCGCAGTGCTGATGA |
| *Pv*gfat-qF | CGACTCTTCTATCTGCCGAGAGTC |
| *Pv*gfat-qR | TCATCTGGGAAGTGTAGGCC |
| *Pv*uap-qF | CAGAGAGGCTGCTGCGACT |
| *Pv*uap-qF | TGGGAGCATTCCTTGTTCG |
| *Pv*ogt-qF | AGAATTGGCCCATCGTGAATAC |
| *Pv*ogt-qR | AGTTGCCCTCGCTCCTTGTA |
| *Pv*oga-qF | GGACTCCAGAACAGCGAAAAGA |
| *Pv*oga-qR | CCAGGGGAAATGGCATAATAAA |
| *Pv*ef1α-qF | TATGCTCCTTTTGGACGTTTTGC |
| *Pv*ef1α-qF | CCTTTTCTGCGGCCTTGGTAG |
| 16S-891-qF | TGGAGCATGTGGTTTAATTCGA |
| 16S-1003-qR | TGCGGGACTTAACCCAACA |
| *Vibrio*-qF | GGCGTAAAGCGCATGCAGGT |
| *Vibrio*-qR | GAAATTCTACCCCCCTCTACAG |
| *V. p*-tdh-qF | GTAAAGGTCTCTGACTTTTGGAC |
| *V. p*-tdh-qR | TGGAATATGAACCTTCATCTTCACC |
| *S. i*-lldP-qF | ACACAGGTGAGCACGCTAAA |
| *S. i*-lldP-qR | CGTCACCATCGTCTTGGTCA |

**Table S2. Potential up-regulated expression of O-GlcNAc-modified proteins under *V. parahaemolyticus* stimulation identified by IP-MS analysis**

Please refer to Supplementary Table S2 for the detailed statistical data presented in the Excel spreadsheet.

**Table S3. Significant DEGs within the glucose uptake–HBP–UDP-GlcNAc module in hemocytes following *V. parahaemolyticus* (VN) or AHPND *V. parahaemolyticus* (VA) challenge**

Please refer to Supplementary Table S3 for the detailed statistical data presented in the Excel spreadsheet.

**Table S4. Summary of mass spectrometry data of the up-regulated O-GlcNAcylation sites on *Pv*HMCL after *V. parahaemolyticus* stimulation**

| **Hemocyanin** | **Modified peptide** | **Position in peptide** | **O-GlcNAcylation sites** | **-10LgP** | **Mass** | **Intensity HMC-PBS** | **Intensity HMC-*V. p*** | **HMC-PBS/HMC-*V. p*** | ***p-value*** |
| --- | --- | --- | --- | --- | --- | --- | --- | --- | --- |
| ***Pv*HMCL** | YALPPGVS(+203.08)DAQK | 8 | Ser**^29^** | 6.01 | 1447.7195 | 587194.2 | 2669291.958 | 0.2199813 | 0.0042741 |
|  | PEADLS(+203.08)HYS(+203.08)DGGAAVQK | 6 | Ser**^67^** | 5.71 | 2149.9651 | 638824.57 | 2144891.627 | 0.2978354 | 0.0240428 |
|  | PEADLS(+203.08)HYS(+203.08)DGGAAVQK | 9 | Ser**^70^** | 5.71 | 2149.9651 | 638824.57 | 2144891.627 | 0.2978354 | 0.0240428 |
|  | S(+203.08)S(+203.08)FT(+203.08)GTKK | 1 | Ser**^191^** | 7.37 | 1463.688 | 13828531 | 43048969.5 | 0.3212279 | 0.035158 |
|  | S(+203.08)S(+203.08)FT(+203.08)GTKK | 2 | Ser**^192^** | 7.37 | 1463.688 | 13828531 | 43048969.5 | 0.3212279 | 0.035158 |
|  | S(+203.08)S(+203.08)FT(+203.08)GTKK | 4 | Thr**^194^** | 7.37 | 1463.688 | 13828531 | 43048969.5 | 0.3212279 | 0.035158 |
|  | EALGGGDSGLENYVSAT(+203.08)GIPNRFLLPK | 17 | Thr**^584^** | 8.84 | 3180.5828 | 3203090.9 | 15262685.78 | 0.2098642 | 0.0031722 |

**Table S5. Average binding energy and dissociation constant for *Pv*HMCL and *Pv*HMCL-O-GlcNAcT584 binding with LPS and PGN**

| Contribution | *Pv*HMCL with LPS | *Pv*HMCL-O-GlcNAcT584 with LPS | *Pv*HMCL with PGN | *Pv*HMCL-O-GlcNAcT584 with PGN |
| --- | --- | --- | --- | --- |
| ΔG_bind_ (kcal/mol) | -8.71 | -10.59 | -10.74 | -11.12 |
| ΔG_final_ (kcal/mol) | -8.89 | -9.89 | -10.76 | -11.36 |
| ΔG_vdW+H_ (kcal/mol) | -8.73 | -9.64 | -10.60 | -11.17 |
| ΔG_elec_ (kcal/mol) | -0.16 | -0.25 | -0.16 | -0.19 |
| ΔG_total_ (kcal/mol) | -8.49 | -9.38 | -10.68 | -10.44 |
| ΔG_tof_ (kcal/mol) | +6.86 | +6.86 | +8.86 | +8.86 |
| ΔG_uns_ (kcal/mol) | -1.81 | -1.81 | -1.81 | -1.81 |
| Kd (nmol/L) | 413.78 | 17.33 | 13.33 | 7.03 |

ΔG_bind_ = Estimated Binding free energy

ΔG_vdW+H_ = van der Waals + Hbond + desolv Energy

ΔG_elec_ = Electrostatic Energy

ΔG_final_ = Final Intermolecular Energy

ΔG_total_ = Final Total Internal Energy

ΔG_tof_ = Torsional Free Energy

ΔG_uns_ = Unbound System's Energyss

Kd = Estimated dissociation Constant. Kd value conversion formula: Kd = ln(ΔG/RT), where R is the universal gas constant with a value of 8.314 J/mol·K, and T represents the temperature in Kelvin (298.15 K).
